# Associations of the placental metabolome with immune maturation up to one year of age in the Swedish NICE-cohort

**DOI:** 10.1007/s11306-024-02092-4

**Published:** 2024-02-26

**Authors:** Olle Hartvigsson, Malin Barman, Hardis Rabe, Anna Sandin, Agnes E Wold, Carl Brunius, Ann-Sofie Sandberg

**Affiliations:** 1 Food and Nutrition Science, Department of Life Sciences, Chalmers University of Technology, Göteborg, Sweden; 2 Institute of Biomedicine, Department of Infectious Diseases, Sahlgrenska Academy, University of Gothenburg, Gothenburg, Sweden; 3 Department of Clinical Sciences, Unit of Pediatrics, Umeå University, Umeå, Sweden

**Keywords:** Placental metabolome, Untargeted metabolomics, Immune maturation, T cells, B cells, Parity, Birth cohort

## Abstract

**Introduction:**

Allergies and other immune-mediated diseases are thought to result from incomplete maturation of the immune system early in life. We previously showed that infants’ metabolites at birth were associated with immune cell subtypes during infancy. The placenta supplies the fetus with nutrients, but may also provide immune maturation signals.

**Objectives:**

To examine the relationship between metabolites in placental villous tissue and immune maturation during the first year of life and infant and maternal characteristics (gestational length, birth weight, sex, parity, maternal age, and BMI).

**Methods:**

Untargeted metabolomics was measured using Liquid Chromatography-Mass Spectrometry. Subpopulations of T and B cells were measured using flow cytometry at birth, 48 h, one, four, and 12 months. Random forest analysis was used to link the metabolomics data with the T and B cell sub populations as well as infant and maternal characteristics.

**Results:**

Modest associations (Q2 = 0.2–0.3) were found between the placental metabolome and kappa-deleting recombination excision circles (KREC) at birth and naïve B cells and memory T cells at 12 months. Weak associations were observed between the placental metabolome and sex and parity. Still, most metabolite features of interest were of low intensity compared to associations previously found in cord blood, suggesting that underlying metabolites were not of placental origin.

**Conclusion:**

Our results indicate that metabolomic measurements of the placenta may not effectively recognize metabolites important for immune maturation.

**Supplementary Information:**

The online version contains supplementary material available at 10.1007/s11306-024-02092-4.

## Introduction

The immune system defends against microbial pathogens, but when dysregulated, it can give rise to both autoimmune diseases and allergies. Immune maturation and downstream protection from allergy development are affected by several lifestyle factors such as growing up with older siblings (Strachan, 1989), pets (Al-Tamprouri et al., 2019), or farm animals (Loss et al., 2011; Jonsson et al., 2016). It has been hypothesized that early life exposure to microorganisms facilitates the development of the immune system, such that it becomes capable of developing active immune tolerance to harmless foreign proteins, which in turn prevents allergy development (Strachan, 1989). Moreover, it has also been shown that the diet consumed by the mother during pregnancy can modulate the risk of allergy development of the child (Jonsson et al., 2016; Barman, 2020, 2021; Calvani et al., 2006; Stråvik et al., 2020; Sausenthaler et al., 2006). During pregnancy, the placenta constitutes the interface between the mother and the fetus, through which nutrients, and other metabolites, and xenobiotics are transferred to the growing fetus. For these reasons, it is reasonable to hypothesize that immune modulation could occur through the transfer of metabolites from the mother to the child via the placenta.

Untargeted metabolomics encompasses the quantitative measurements of small molecules (usually below 1500 Da) in a biological sample (e.g., urine, plasma, and tissue). Liquid chromatography-mass spectrometry LC-MS provides the largest coverage of the metabolome of the methods available and can reflect, e.g. metabolic pathway regulation, as well as exposures such as diet or pollutants (Ulaszewska et al., 2019).

We have recently discovered an association between the metabolite profile of umbilical cord blood and the fraction of B cells in the infant’s circulation displaying a memory phenotype, suggesting that certain bioactive molecules produced in the pregnant woman may affect the immune maturation of the infant (Hartvigsson et al., 2021). Previous studies have observed associations between the placental metabolome and pre-eclampsia (Dunn et al., 2009), spontaneous preterm birth (Elshenawy et al., 2020), and fetal growth restriction (Bahado-Singh, 2020). We, therefore, asked whether the placental metabolome could be linked to immune maturation in the fetus and infant. However, to the best of our knowledge, no studies have investigated associations between the placental metabolome and infant immune maturation.

Therefore, we aimed to investigate associations between the placental metabolome and subpopulations of T and B cells sampled at five time points from birth to one year of age using an untargeted approach. Further, in a directed analysis, we investigated whether metabolite features of maternal and infant blood previously linked to infant immune maturation (Hartvigsson et al., 2021) would also be reflected in the placental metabolome. In addition, we investigated to what extent the placental metabolome reflected neonatal and maternal traits such as gestational length, the infant’s birth weight and sex, and the mother’s parity, age, and BMI.

## Materials and methods

### Study population

The birth-cohort NICE (Nutritional impact on the Immunological maturation during Childhood in relation to the Environment) recruited 655 pregnant women during 2015–2018 with planned delivery at the Sunderby Hospital, in northern Sweden. More details about the cohort can be found in the study protocol (Barman et al., 2018) and previous papers from the cohort (Barman, 2021; Stråvik et al., 2020; Hartvigsson et al., 2021). The inclusion criteria for the NICE-cohort were to have a planned delivery at Sunderby Hospital and to be able to understand written and oral instructions in Swedish.

The study was approved by the Regional Ethical Review Board in Umeå (2013-18-31 M, 2016-232-32); written informed consent was obtained from the prospective parents. The study was performed in accordance with the ethical approval and in accordance with the declaration of Helsinki.

Placentas were collected at delivery and used for this study if processed within 4 h after delivery, resulting in a total of 96 placental samples analyzed. The clinical characteristics of the 96 newborn infants and their mothers who were included in the study are shown in Table 1.

**Table 1:** Clinical characteristics for the study participants (*N* = 96)

Characteristics	Median (25th – 75th percentile) or n (%)
Gestational length (days)	279 (275–286)
Birth weight (g)	3605 (3350–3992)
Sex (Female)	51 (53%)
Parity (> 0)^a^	53 (55.2%)
Caesarean section	11 (11.5%)
Maternal age (years)^b^	29 (26.5–34)
Maternal BMI (kg/m^2^)^b^	24.1 (21.3–27.8)

^a^Defined as either nulliparous or parous

^b^Assessed at admission to maternity clinics in the first trimester.

### Placenta sampling

**Fig. 1:**
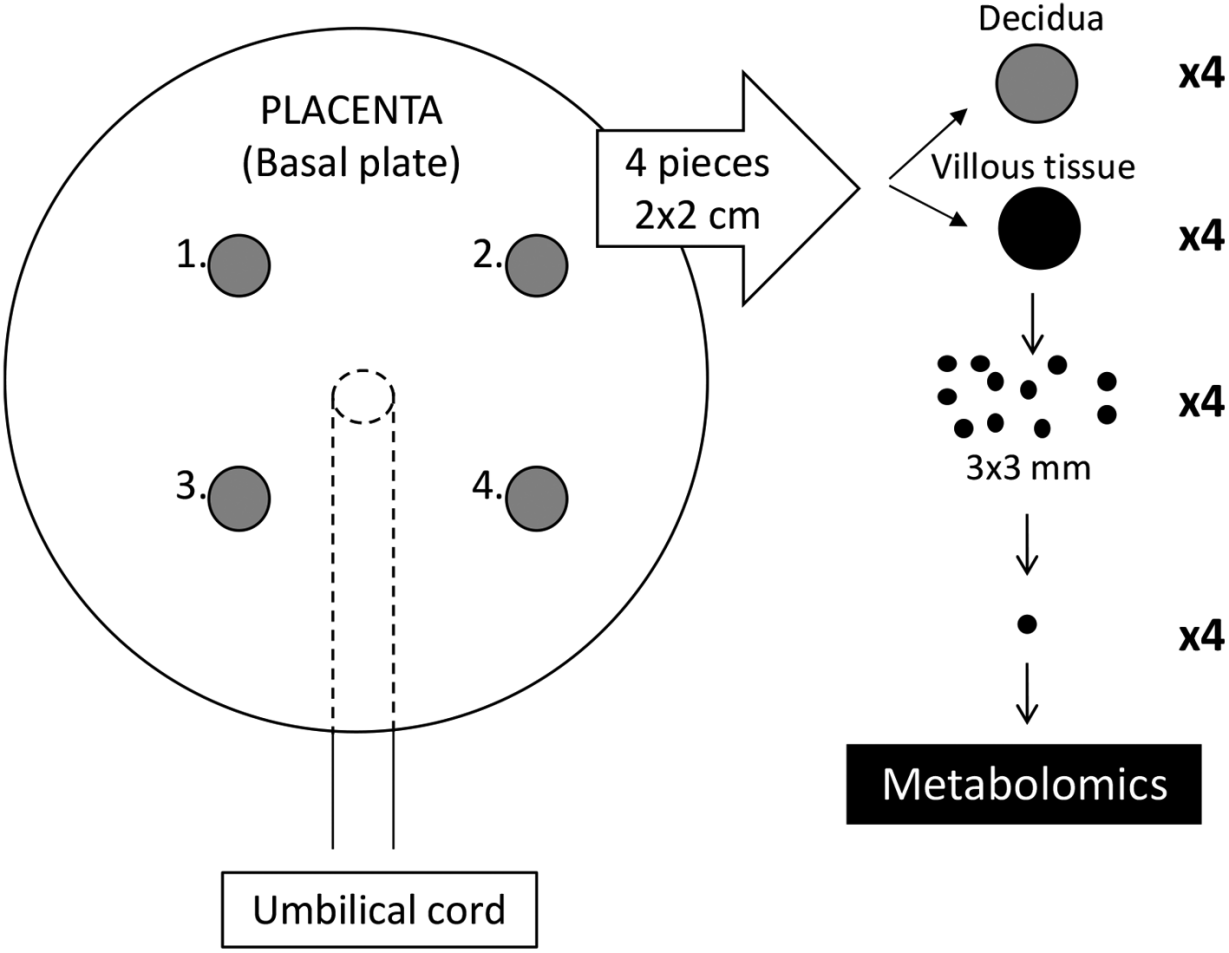
Sampling of chorionic villous tissue from the placentas. Small pieces, 3 × 3 mm of chorionic villous tissue, were sampled from 4 parts of the placenta for metabolomic measurements

Four portions of chorionic villous placental tissue (1–2 cm^3^), were cut out from four places on each placenta, aiming at a similar distance from the cord insert. Fetal and maternal sides (i.e., chorionic plate and decidua) were removed (Fig. 1). The pieces of villous tissue samples were washed twice in ice-cold phosphate-buffered saline to remove contaminating blood. Each piece was further cut into small pieces 3 × 3 mm, which were dispersed into micro tubes and stored at -80 °C. Other parts of the placenta were collected for other purposes as described in detail in the NICE-cohort study protocol (Barman et al., 2018).

### Sampling of blood during pregnancy and mother’s and infant’s cord blood at delivery

Maternal venous blood was sampled in gestational week 28 and during delivery by venipuncture into EDTA tubes. Mothers were encouraged to fast in connection to the visit at gestational week 28 but not in connection to delivery. Infant cord blood was sampled, as described elsewhere (Hartvigsson et al., 2021), by squeezing the remaining blood from the severed umbilical cord into EDTA tubes. Samples were left at room temperature for 30 min before centrifugation and stored at 4 °C until aliquoted and frozen at -80 °C. In total, 605 maternal blood samples from pregnancy, 558 from delivery, and 366 infant umbilical cord blood samples were obtained.

### Metabolomics analysis

Sample preparation and analytical procedures were based on procedures developed by Hanhineva and colleagues (Pessa-Morikawa et al., 2022). One piece from the four placental replicates was analyzed separately in random order in 5 analytical batches. Aliquots of 100 mg per piece were cryo-ground using a Retsch mixer mill 400 (Sigma-Aldritch), using 5 mm stainless steel beads in precooled 2 × 24 adapters shaken for 2 min at 30 Hz. To the ground samples, 10 µl methanol (90% v/v H2O, LC-MS Ultra CHROMASOLV®, Fluka) per mg sample was added. Samples were then shaken (20 min, 4 °C) followed by centrifugation (13000 rpm, 10 min, 4 °C) and filtration (Captiva ND plates; Agilent P/N A5969045) using water vacuum for 5 min. Before analysis, the samples were further diluted tenfold with 90% (v/v) methanol. After centrifugation (500×g, 1 min, 4 °C), 45 µl per sample was transferred to a UHPLC vial (Amber 51, 2 mL, Phenomenex), one each for reversed-phase and HILIC untargeted LC-MS metabolomics. Study-specific QC samples were made by pooling 25 µl from each sample in the first batch.

Untargeted LC-MS metabolomics was performed on an Agilent 1290 UHPLC coupled to an Agilent ESI iFunnel qTOF 6550 mass spectrometer. Centroided MS data was collected with MassHunter B.08.00. LC and MS settings are reported in Suppl. Table S1. Five QCs were injected before the first sample for each batch and after every 12 samples.

### Preprocessing

Peak picking, retention time adjustment, and peak correspondence for the placenta samples were performed using MS-Dial version 4.24. The peak picking and alignment parameters can be found in Suppl. Table S2. From this point, all computations were made using R version 3.6.0 (Core Team, 2019). Intensity drift was adjusted using batchCorr v 0.2.4 (Brunius et al., 2016). Principal Component Analysis indicated data sanity (Suppl. Fig. S1). Isotopes, adducts, and fragments were aggregated using RamClust v1.0.6 (Broeckling et al., 2014). Feature quality and reproducibility were determined using the replicate samples by setting up a criterion of feature CV < 30% in at least 50% of the quadruplicates. This criterion on within-sample analytical stability resulted in retaining more analytically stable features measured by CV_QC_ whilst retaining between-sample variability (Suppl. Figs. S2 and S3). Replicates were then averaged for each remaining feature resulting in a total of 4664, 2681, 860, and 795 features for reverse phase positive (RP), reverse phase negative (RN), HILIC positive (HP), and HILIC negative (HN), respectively. The number of features at each key point of the preprocessing can be found in Suppl. Table S3.

### Flow cytometry

Flow cytometry was performed on blood samples collected from infants at birth (cord blood) (n = 32), 48 h (n = 21), one month (n = 22), four months (n = 25), and one year (n = 27) of age, as described previously (Hartvigsson et al., 2021). Due to the difficulty of obtaining blood from small infants, immune cell analyses were only available for a subset of the infants from whom we had placenta samples available. A full list of investigated cell types, along with the number of samples for each measurement, is found in Suppl. Table S4. Samples were stored dark at room temperature directly after collection, and staining was performed within 48 h of sampling. Whole blood (50 µl) was added to TruCount™ tubes (BD Bioscience, Erembodegem, Belgium), together with 20 µl antibody cocktail containing anti-CD4, anti-CD8, anti-CD20 and anti-CD45 (Suppl. Table S5) and incubated dark at RT for 15 min. BD Lysing Solution was added and allowed to act for 15 min. Samples were analyzed within 1 h of staining in an Accuri C6 (BD Bioscience). For each sample, 5000 beads were collected in the flow cytometer.

For T and B cell phenotypes, 900 µl blood was lysed with RBC lysis buffer (eBioscience) (15 min, RT) and stopped with FACS buffer before centrifugation (5 min, 300×g). The supernatant was discarded, and the pellet was resuspended in 1 ml FACS buffer. Cell suspensions (50 µl) were stained with 30 µl antibody cocktail (Table S4) in 96 V-bottom plates for (20 min, 4 °C, dark), washed with 300 µl FACS-buffer, and centrifuged (3 min, 300×g). The supernatant was discarded, and the cells were resuspended in 300 µl buffer (Foxp3 Fixation/Perm. Kit, eBioscience) and incubated at room temperature for 15 min. Cells were then centrifuged (3 min, 500×g), washed with Foxp3 buffer, again centrifuged (3 min, 500×g), resuspended in FACS-buffer and stored in the dark (4 °C) until analysis in the Accuri C6 flow cytometer. Flow cytometry data were analyzed using Flow Jo v10 (TreeStar, Ashland, Oregon).

### PCR analysis for KREC and TREC

Blood samples were collected in EDTA-tubes from the children at birth (cord blood), 48 h after delivery, 1, 4 and 12 months of age. For analysis of T-cell-receptor excision circles (TREC) and Kappa-deleting element recombination circle (KREC), 500 µl blood collected from all children participating in the cohort, was alliquoted into 1 ml tubes and frozen at 80oC until analysis. Genomic DNA was extracted from 200 µl whole blood using QIAamp DNA Blood Mini Kit (Qiagen, Cat No. 51106) as described by the manufacturer. Purified DNA concentrations were assessed fluorometrically (Quantus®, Promega) and adjusted to 30 ng/µL with DNase/ RNase free H2O (Gibco, no. 10977). Triplex real-time PCR reactions were performed in duplicate in a Roche Light Cycler 480 II instrument to detect the number of copies of TRECs, KRECs and ACTB (beta Actin) as control gene. The following reagents were used: LightMix® Modular TREC, LightMix® Modular KREC, LightMix® Modular Actin Extraction Control (ACTB) all from TIB MOLBIOL Syntheselabor GmbH and Roche. The PCR-mix contained 0,5 µL from every above mentioned reagent mix, 5 µL sample or controls (dH2O as negative control and 30 ng/µl cord blood as positive), 9,5 µL PCR-grade H2O and 4 µl master mix (Roche LightCycler® Multiplex DNA Master) for a total of 20 µl. Results for every sample were calculated by extrapolating the absolute concentration from standard curves constructed with known dilutions (106 to 101) of genes of interest (Standard Row TREC & KREC (genomic) 30–621/622), with fixed concentrations of TREC, KREC (103) and ACTB (3 × 104) as curve adjustors. The formula (TREC or KREC) copies) x106/ (½*ACTB copies) was used to determine the concentration of TRECs or KRECs in 106 white blood cells.

### Data analysis

We did not expect associations between response and outcome variables to necessarily be linear, and thus investigated associations using Random Forest analysis. To associate the placental metabolome with the T and B cell subpopulations measured at birth, 48 h, one, four, and twelve months of age (all measured subpopulations are reported in Suppl. Table S4) Random Forest regression was performed in a repeated double cross-validation to reduce false positive discovery using the MUVR package v 0.00.971 (Shi et al., 2018). Furthermore, this method performs an unbiased selection of variables-of-interest underlying the association. Significance was assessed using permutation tests (n = 200) on models with potentially relevant performance, defined a priori as Q2 values > 0.2 (chosen to reflect meaningful predictions). In brief, for all models with Q2 > 0.2, RF regression was conducted 200 times using the same independent variables but with a permuted dependent variable. Q2 values from these permuted models were extracted into a null hypothesis distribution and a p-value of the original model was calculated as the cumulative probability of observing the actual model’s Q2 in the null hypothesis distribution represented as a t-distribution. Features selected by the MUVR algorithm were further associated with its relevant outcome using spearman correlation.

Metabolomics analyses in plasma were performed similar to the placenta metabolome analysis described, however, using acetonitrile instead of methanol as an extraction solvent, and are described in detail elsewhere (Hartvigsson et al., 2021). Features in maternal and umbilical cord blood plasma that we previously observed to associate with immune maturation during infancy (Hartvigsson et al., 2021) were searched for based on the mass-to-charge ratio in the placental metabolome and investigated for associations with the immune parameters. Moreover, features associated with the placental metabolome in this study were also investigated in maternal and umbilical cord blood plasma. This was achieved by searching for features of the same m/z and rt with a mass difference tolerance of 20 ppm and a retention time window of ± 30 s.

Furthermore, associations between the placental metabolome and several maternal and neonatal traits, including gestational length, the infant’s birth weight and sex, the mother’s parity, age, and BMI, were investigated. This was achieved by random forest regression as described above as well as random forest classification for the dichotomous variables sex and parity, with the addition of permutation testing (n = 200) for each outcome.

### Metabolite annotation

Features of interest were selected from the MUVR-RF algorithm, and these features were further analyzed by targeted MS2. Vendor format files were converted to mgf and mzML format using the Proteowizard MSconvert GUI (Chambers et al., 2012). Annotation was performed using first matching against an in-house library of standards (built from the MSML standard kit, Iroa Technologies, thus representing general metabolomics purposes), followed by matching to the MONA-database (in-house script available upon request). Further, spectral data from both the mgf and mzML files were loaded into Sirius (Dührkop et al., 2019), to get further information about the features using a combination of CSI:fingerID (Hoffmann, 2021; Dührkop et al., 2015) and CANOPUS (Dührkop, 2020; Feunang et al., 2016). Annotation confidence of metabolites are reported as suggested by the Metabolomics Standards Initiative (MSI) (Sumner et al., 2007).

## Results

### Immune cell development during the first year of life

**Fig. 2:**
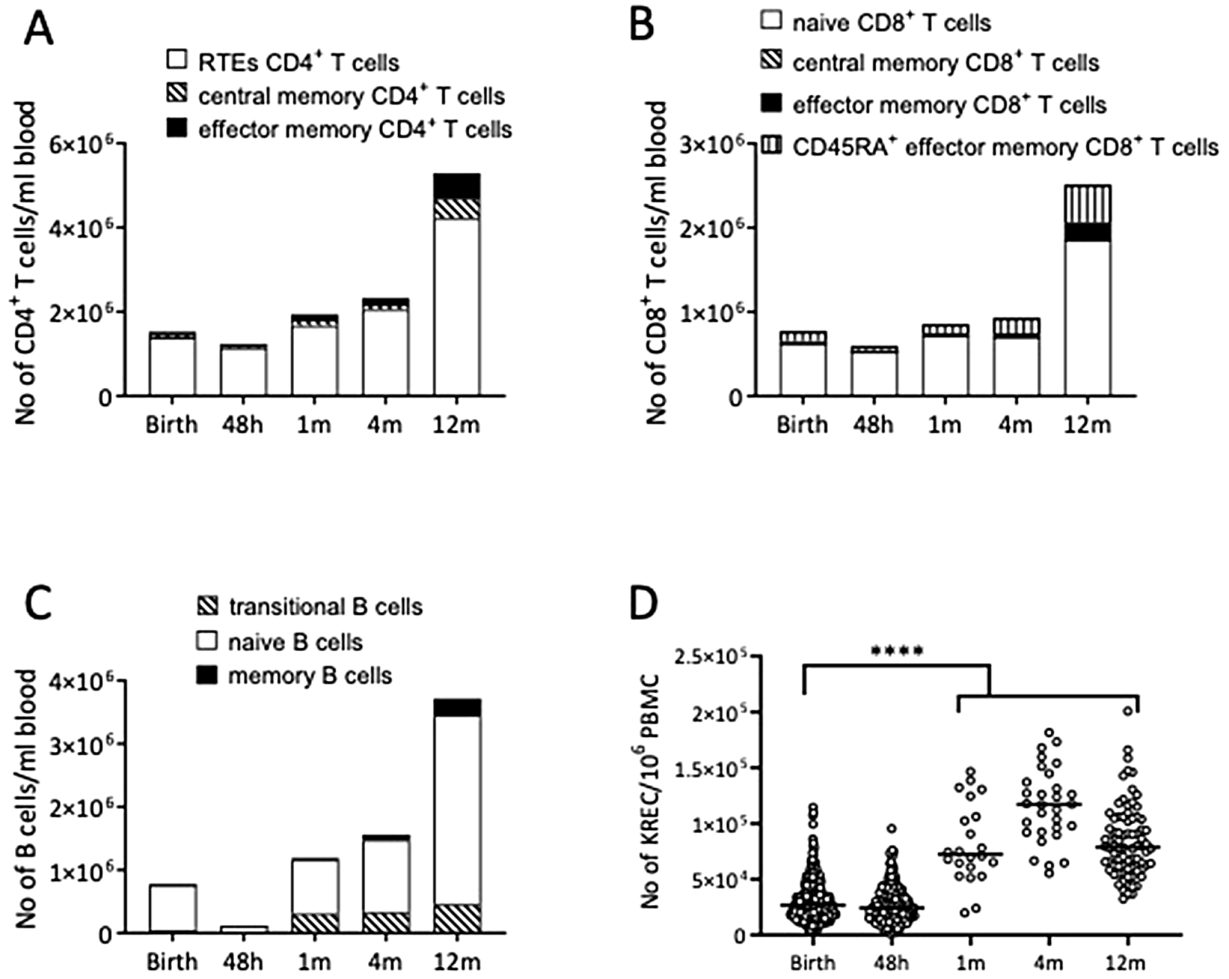
Blood samples collected at birth, 48 h, 1, 4 and 12 months after birth were analyzed for the numbers of different lymphocyte populations with the use of flow cytometry, as well as the number of KREC by qPCR. (A-C) The median number of transitional, naïve CD24 + CD38 + and CD27 + memory B cells (A), RTE (recent thymic emigrants), CCR7 + CD45RA- central memory and CCR7-CD45RA- effector memory T cell CD4 + T cells (B) as well as CCR7 + CD45RA + naïve, CCR7 + CD45RA- central memory, CCR7-CD45RA- effector memory T cell and CCR7-CD45RA + effector CD8 + T cells (C), during the first year in life. (D) The numbers of circulating KREC per 10^6^ PBMC during the first year of life. Each dot represents one child, and the horizontal bar represents the median value. Mann Whitney’s U test, ****p < .00001

In the NICE study, the number of lymphocytes, i.e. B-, CD4 + T cells and CD8 + T cells, increased during the first year in life (Fig. 2A-C). Furthermore, the proportion and number of memory cells, i.e. CD4 + CD38 + memory B cells, CCR7 + CD45RA- central memory T cells of both CD4 + and CD8+, as well as the CCR7-CD45RA- effector T cell subsets increase from 1month of birth up to 12 months of life. These data might suggest a maturity of the immune system during the first year of life.

### Placental metabolic profiles associated with immune maturation during the first year of life

Associations were observed between the placental metabolome and four different immune parameters. We observed that the number of Kappa-deleting recombination excision circles (KREC) at birth was associated with the metabolomic profile of the placenta.

Further, the fraction of B cells displaying a naïve phenotype at 12 months of age (CD24lowCD38low) was associated with the placenta metabolome. Regarding T cells, the number of CD4 + T cells with the memory phenotype (RA-), as well as the fraction of CD4 + T cells expressing the memory phenotype at four months of age (Table 2), were associated with the placental metabolome. No associations were observed relating to any of the measured immune cell populations at 48 h or one month. Q2 values from all analyses available in Suppl. Table S4.

**Table 2:** Associations of placental metabolome and immune maturation

Cell population	N	Q2	p value^a^
KREC at birth	36	0.33	0.003
Proportion of CD24low CD38low naïve B cells of total B cells at 12 months	23	0.26	0.006
Number of CCR7 + CD45RA- of CD4 + T cells at 4 months	22	0.23	0.028
Proportion of CCR7 + CD45RA- of CD4 + T cells at 4 months	25	0.26	0.002

^a^p values from permutation analysis (n = 200)

The metabolite features that contributed to the placental metabolome and immune maturation associations (Table 2) are shown in Table 3. Most of the features of interest were obtained from reverse-phase chromatography with positive ionization. A majority of the features were present in low intensities, but all were considered true peaks upon manual inspection (Suppl. Table S7).

KREC levels in blood leukocytes were positively associated with one metabolite and negatively associated with four metabolites, whilst the proportion of B cells that displayed a naïve phenotype at 12 months of age was positively associated with five features and negatively associated with five features. The blood concentration of CCR7 + RA- CD4 + T cells at four months was positively associated with seven features and negatively associated with one feature, and the fraction of CD4 + T cells that displayed the CCR7 + RA- markers at four months was positively associated with six features.

None of the features of interest found to associate with either outcome could be identified (i.e., MSI annotation level 4), mainly due to low intensity, effectively making MS2 spectra unattainable.

**Table 3:** Features of interest associated with immune maturation measurements from models with Q2 > 0.2, presented with Spearman correlation coefficient and p-value from correlation test

Feature (m/z@rt)	Rho	p-value
*KREC in umbilical cord blood*		
RP285.27487@418.98	-0.55	< 0.001
RP579.18646@327.72	-0.46	0.005
RP279.07724@362.7	0.60	< 0.001
RP511.27478@207.3	-0.27	0.1
HN112.9861@343.62	-0.31	0.06
*Proportion of CD24low CD38low naïve B cells of total B cells at 12 months*		
HN329.2338@52.742	-0.74	< 0.001
RP78.03462@76.56	0.71	< 0.001
RP379.13776@185.76	-0.48	0.021
RP175.15396@325.68	-0.47	0.024
RN568.79736@32.942	0.52	0.011
HN92.05019@54.42	-0.61	0.002
RP319.14124@170.4	-0.54	0.008
RN196.94266@36.36	0.62	0.002
RN746.80267@35.882	0.65	< 0.001
RP274.87427@35.822	0.63	0.001
*Number of CCR7 + CD45RA- T cells at 4 months*		
RP332.25632@272.64	0.63	< 0.001
RP386.29367@329.64	0.69	< 0.001
RP358.26218@296.10	0.68	< 0.001
RP464.33435@365.28	0.76	< 0.001
RP228.08873@116.88	0.59	0.002
RP442.37125@366.12	0.69	< 0.001
HP422.10547@368.64	-0.65	< 0.001
RP360.28867@312.00	0.64	< 0.001
*Proportion of CCR7 + CD45RA- T cells of CD4 + T cells at 4 months*		
RN596.4267@434.762	0.65	< 0.001
RN245.07942@348.66	0.74	< 0.001
RN381.16165@455.22	0.78	< 0.001
RP273.10336@39.84	0.61	0.002
RN145.03938@121.62	0.67	< 0.001
RN417.17871@443.58	0.69	< 0.001

Feature clusters generated by RamClustR, full info about which features are included in which cluster available in Suppl. Table S6. Abbreviations: RP – reversed phase, Positive ionization, RN – reversed phase negative ionization, HP – HILIC positive ionization, HN – HILIC negative ionization; retention time in seconds

### Overlap of immune-related features between the placenta and maternal and cord blood plasma

None of the immune-related metabolites in the placenta could be found in the measured cord and maternal plasma used in our previous study. Vice versa, none of the previously reported immune-related plasma metabolites could be found in the placenta metabolome.

### Placental metabolic profiles associated with neonatal and maternal traits

Among the neonatal and maternal traits investigated (gestational length, birth weight and sex of the infant, maternal parity, age, and BMI), only infant sex and parity were associated with the placental metabolome, and the classification rates were modest (Table 4). This was confirmed by associating traits to principal components of the overall metabolome, showing only weak associations (Suppl. Figure S4).

**Table 4:** Performance when associating the placental metabolome with the neonatal and maternal factors gestational length, birth weight, sex, parity, maternal age, and maternal BMI

Outcome	N	Q2 or classification rate	p-value
Gestational length	96	-0.12	0.228
Birth weight	95	-0.06	0.349
Maternal age	96	-0.19	0.261
Maternal BMI	94	-0.02	0.132
Sex	96	65%	0.033
Parity	96	65%	0.048

Regression models were performed for continuous and classification models for dichotomous outcome variables

## Discussion

This study aimed to investigate associations between the placental metabolome and immune maturation in infants measured as concentration and proportion of certain subpopulations among T and B cells and TRECs and KRECs. We observed associations of the placental metabolome with the KREC content of the umbilical cord blood lymphocytes, with the proportion of B cells having a naïve phenotype at four months of age and with memory T cells at one year of age. However, none of the metabolites selected by the multivariate analysis to be of interest underpinning these associations could be identified.

The strongest, albeit still modest, association observed between the placental metabolome and immune cells was to the number of KRECs in the umbilical cord blood, which reflect B cells formed in the bone marrow that have not undergone clonal expansion during immune responses (31). In addition, associations were observed between the placental metabolome and the proportion of CD24low CD38low naïve B cells at one year of age. Naïve B cells have not yet encountered their corresponding antigens and represent the majority of the B cells in the newborn infant but decrease in proportions as the child ages (32). These results indicate an association between the placental metabolome and either the production of naïve B cells in the bone marrow or, conversely, their proliferation in response to antigen (which reduces their KREC content and converts them into a memory phenotype). Also, the placental metabolome was associated with the number and proportion of CCR7 + CD45RA- T cells at one year of age, representing memory T cells (33). Memory T cells are formed after a naïve T cell has encountered its corresponding antigen and remains in the body long after the antigen that activated the T cell is gone, causing a faster reaction to the same antigen upon new exposure.

Collectively, these associations, although modest, suggest that metabolites may be involved not only in fetal immune function but also that they might have a long-term effect on the reaction of the infant’s immune system, possibly through an effect during fetal life. This is supported by our previous results, where we observed associations between metabolites in maternal and umbilical cord plasma and immune maturation markers (11).

However, the results do not suggest the origin of such metabolites. In fact, since most metabolite features associated with the measured outcomes in the present study are of low abundance, this could suggest that the metabolites driving these associations might not originate from the placenta but rather from the mother and only pass by the placenta, making the placenta a passive carrier of the metabolites behind the immune maturation mechanisms, rather than the organ itself being involved in it. Alternatively, if these metabolites originated from the placenta, they are either rapidly cleared, produced in very low concentrations, or poorly detectable by the employed analytical protocol.

In examining potential cross-talk between the plasma and placental metabolomes, none of the previously reported immune-related plasma metabolites (11) could be detected in the placental metabolome or vice versa. This lack of overlap strengthens the notion that the placenta might not be a suitable matrix for identifying metabolites that modulate the immune maturation of the infant and that possible immunomodulating metabolites are neither generated nor enriched in the placenta. Another potential explanation could be that acetonitrile was used as a solvent when extracting metabolites from the plasma samples, whereas methanol was used in the present study, most notably affecting the extraction of lipid species. However, we cannot exclude the possibility that the findings in this or our previous study (12) reflect spurious associations and can thus not be replicated. Future studies investigating the role of the placenta in immune maturation and allergy could consider increasing the metabolome and lipid coverage, e.g., by employing biphasic extraction. In addition, other potential omics strategies, such as proteomics and transcriptomics, could reveal other aspects of metabolic regulation to investigate the role of the placenta in immune maturation.

Interestingly, the placental metabolome associated only weakly with the sex of the child and the mother’s parity status and not with any of the other measured traits. These results are surprising since previous studies have reported sex-specific differences in placental gene expression and metabolites (34–36). Also, our previous study showed parity-related differences in the venous umbilical cord metabolome (37). The present results suggest that the placental metabolome appears to contain less (or less clear) information about general traits as well as immune maturation compared to umbilical cord plasma.

A limitation of our study is the limited sample size, as only 96 placentas were considered suitable for analysis, and not all of these had corresponding flow cytometry measurements; However, given the lack of well-defined study material and the consequent scarcity of studies involving placenta metabolomics and immune maturation, we consider the results from this exploratory study important. Furthermore, even though the children of the study were followed over time, not all the available flow cytometry measurements were obtained from all children at all time points, making longitudinal analysis unfeasible since it would further decrease statistical power. However, we caution that the results should be interpreted with care. The lack of metabolite annotation remains a major limitation: Most of the features of interest were in low abundance and, therefore, difficult to characterize. In addition, features where MS2 spectra were obtainable, did not yield any plausible spectral matching to in-house or online reference databases, nor using the Sirius tool. Annotation is indeed a major bottleneck in metabolomics studies in general, and since the placenta has not been an extensively studied material for metabolomics, even less information is available, further aggravating this issue.

## Conclusions

We observed modest associations between the placental metabolome and the development of some immune cell types (newly formed B cells, naïve B cells, and memory T cells). Furthermore, only weak associations could be observed between the placental metabolome and the parity and sex traits. None of the metabolites underpinning the observed associations could be identified, mostly due to low abundance but also due to a lack of spectral matches. The general low intensity could suggest that these metabolites were not of placental origin and that the placenta may not be an optimal matrix for metabolomics studies on the maturation of the immune system, nor the effect of maternal and infant traits.

**Supplementary Materials**: Figure S1. PCA showing biological and Quality Control (QC) samples for a visual assessment of the analytical variance. Figure S2. Histograms showing the coefficient of variation (CV) of QC samples for all features, features that were kept for subsequent analysis and features that were removed due to too high within-sample variability. Features removed due to high inter-sample variability also showed higher analytical variability. Figure S3. Scatterplots showing the coefficient of variation (CV) for samples (y-axis) and QCs (x-axis) for all features, features that were kept for subsequent analysis and features that were removed due to too high within-sample variability. Features removed due to high inter-sample variability also showed higher analytical variability, and a high degree of between-sample variability. Figure S4. PCA of all samples with Spearman correlation coefficients between PC scores and infant and maternal traits superimposed. Table S1: LC and MS settings used for the analysis of placenta samples.; Table S2: Final parameters for preprocessing using xcms and RAMClust; Table S3: Number of metabolomic features per LC-MS mode at key steps during preprocessing; Table S4: All subpopulations of T and B cells investigated presented together with the number of samples and Q2 from each multivariate model; Table S5: Antibodies used in the flow cytometry analysis; Table S6: All features belonging to clusters that showed to associate with significant outcomes. Table S7. Evaluation of Peak Quality (Table and Extracted Ion Chromatograms).

### Electronic supplementary material

Below is the link to the electronic supplementary material.

Supplementary Material 1

## Data Availability

The data presented in this study are available on request from the corresponding author. The data are not publicly available due to ethical restrictions.
