# Supplementary material for: Associations of the placental metabolome with immune maturation up to one year of age in the Swedish NICE-cohort
Source: Metabolomics. 2024 Feb 26;20(2):28. doi: 10.1007/s11306-024-02092-4 (PMC10896773; doi:10.1007/s11306-024-02092-4)

Supplementary material

Associations of the placental metabolome with immune maturation up to one year of age in the Swedish NICE-cohort

Olle Hartvigsson[1], Malin Barman[1*], Hardis Rabe[2], Anna Sandin[3], Agnes E Wold[2], Carl Brunius[1], Ann-Sofie Sandberg[1]

[1]Food and Nutrition Science, Department of Life Sciences, Chalmers University of Technology, Göteborg, Sweden;

[2] Institute of Biomedicine, Department of Infectious Diseases, University of Gothenburg, Gothenburg, Sweden;

[4] Department of Clinical Sciences, Unit of Pediatrics, Umeå University, Umeå, Sweden.

*Correspondence: malin.barman@chalmers.se.

Supplementary methods

Feature filtering

Intensity filtering was performed by setting an intensity threshold for the highest integrated area of each feature, based on instrument experience of intensity required for obtaining MS2 spectra. The selected intensities for filtering were 100000, 50000, 100000 and 50000 for RP, RN, HP and HN respectively. Further, as several peaks likely corresponding to dead volume were selected, an additional filtering step was performed where features with an m/z of >300 and a retention time of <60s were filtered out for noise removal. After this filtering, a total of 2563, 1459, 505 and 504 features remained for RP, RN, HP and HN respectively.

Supplementary figures

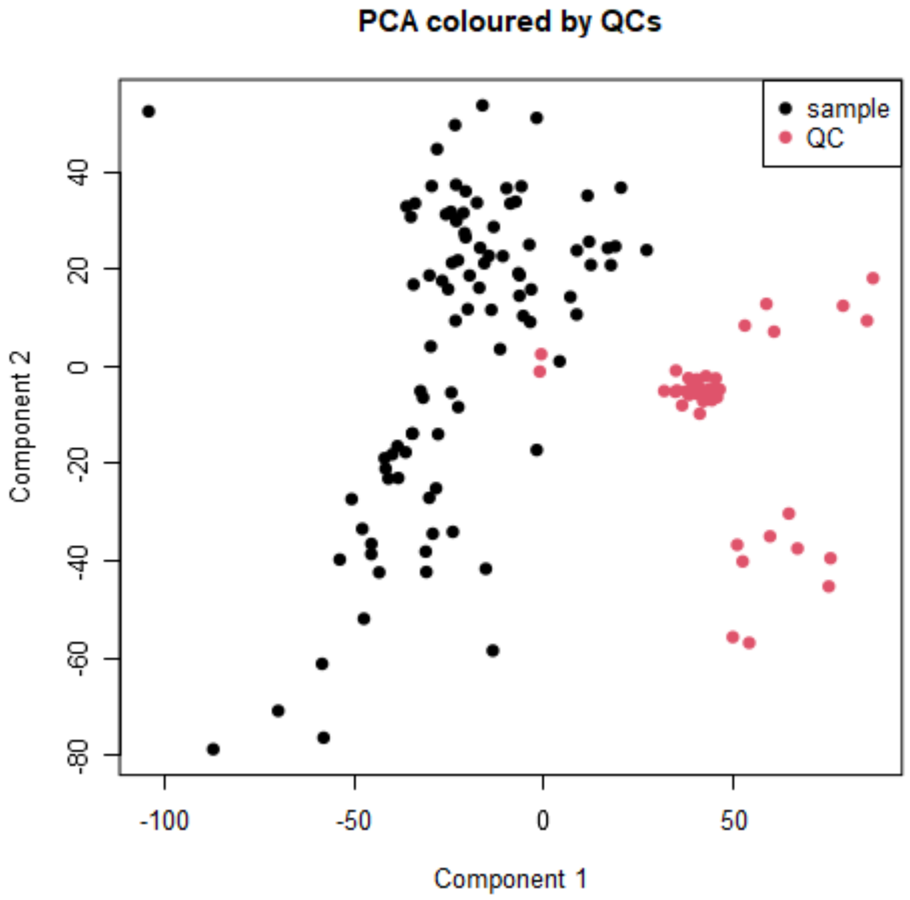

Figure S1. Principal Component Analysis (PCA) showing biological and Quality Control (QC) samples for a visual assessment of the analytical variance.

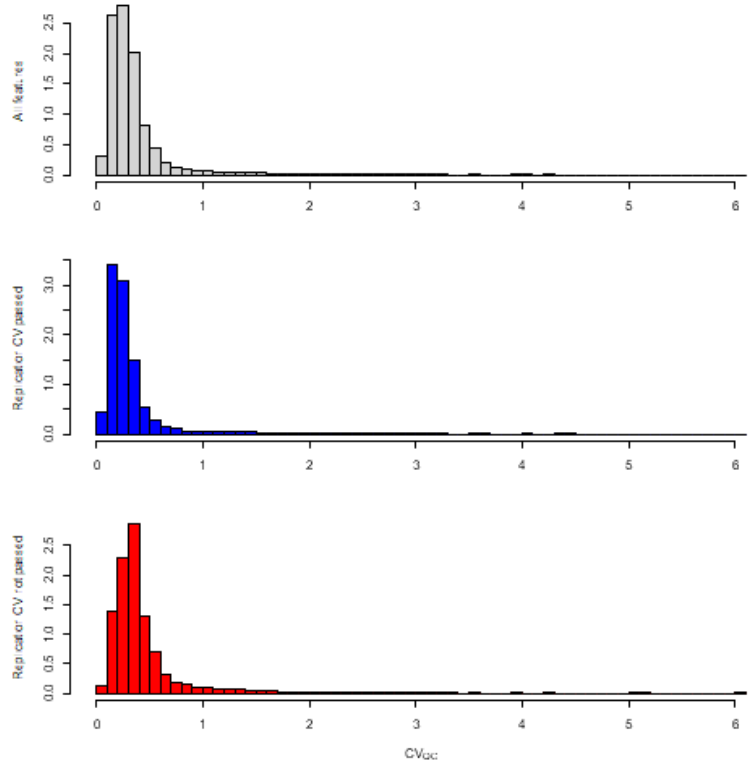

Figure S2. Histograms showing the coefficient of variation (CV) of QC samples for all features, features that were kept for subsequent analysis and features that were removed due to too high within-sample variability. Features removed due to high inter-sample variability also showed higher analytical variability.

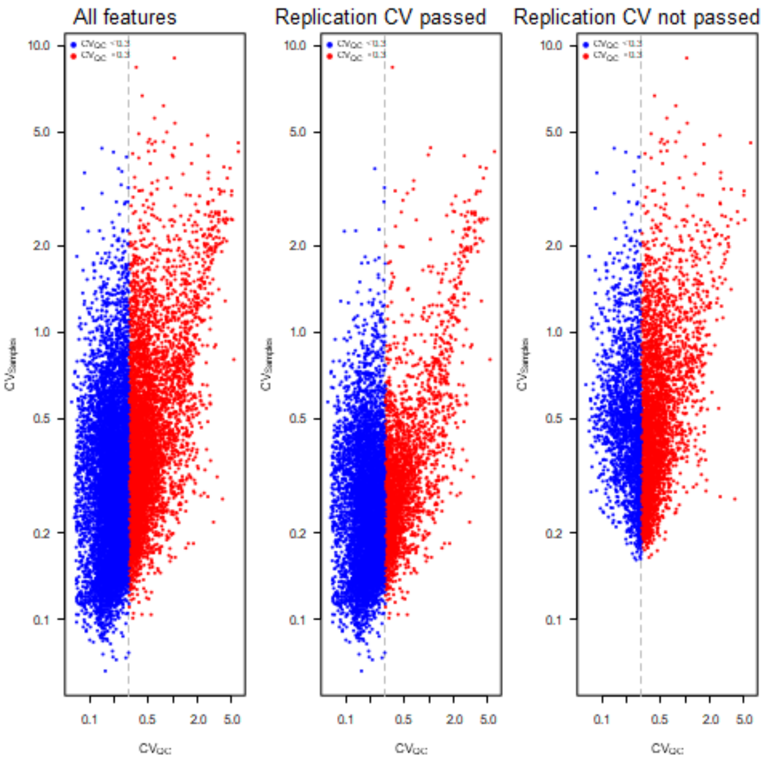

Figure S3. Scatterplots showing the coefficient of variation (CV) for samples (y-axis) and QCs (x-axis) for all features, features that were kept for subsequent analysis and features that were removed due to too high within-sample variability. Features removed due to high inter-sample variability also showed higher analytical variability, and a high degree of between-sample variability.

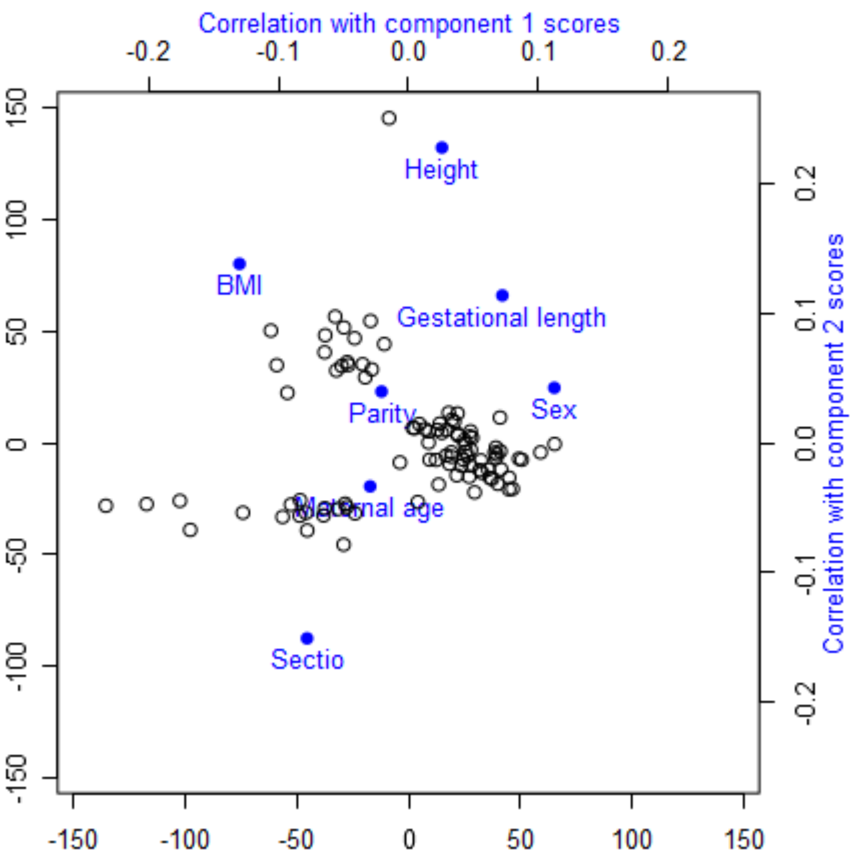

Figure S4. PCA of all samples with Spearman correlation coefficients between PC scores and infant and maternal traits superimposed.

Supplementary Tables

Table S1. LC and MS settings used for analysis of placenta samples.

LC and MS settings		
**Setting/equipment**	**Reversed phase**	**HILIC**
Column	Waters Acquity UPLC HSS T3, 100 x 2.1 mm, 1.8 μm	Waters Acquity BEH Amide 100 x 2.1mm, 1.8 μm
gradient	5%MeOH-95% water, ramped up to 100 % MeOH over 6 min and held for 4.5 min. Both solutions with 0.04 % formic acid	100 % MeCN, held for 1 min. Ramped to 30/70 (water/MeCN) over 7 min. Both solutions (water and MeCN) containing 10mM ammonium formate
Sample volume	3 μL	3 μL
Column temperature	45 °C	45 °C
Flow rate	0.4 mL/min	0.4 mL/min
Ionization source	Dual ESI	Dual ESI
Scanning range	50-1700 m/z	50-1700 m/z
Scanning rate	1.67 spectra/second	1.67 spectra/second
Capilary voltage	3500 V	3500 V
Gas temperature	175 °C	175 °C
Gas flow	10 L/min	10 L/min
Nebulizer	45 psig	45 psig

Table S2. Final parameters for preprocessing using xcms and RAMClust.

MS-Dial peak picking parameters				
**Analytical mode**	**RP**	**RN**	**HP**	**HN**
MS1 tolerance	0.03	0.05	0.025	0.03
Minimum peak width	5	5	5	5
Minimum peak height	3000	1000	3000	3000
Retention time tolerance	0.08	0.08	0.07	0.1

Table S3. Number of metabolomic features per LC-MS mode at key steps during preprocessing.

Step	RP	RN	HP	HN
MS-Dial	27227	29996	4689	3682
BatchCorr	11137	10542	2427	2261
RamClust	7333	4569	1511	1257
Feature stability assessment	4664	2681	860	795

Table S4. All subpopulations of T and B cells investigated presented together with number of samples and Q2 from each multivariate model.

Cell type	n	Q2
TREC, Birth	36	-0.33
KREC, birth	36	0.27
No CD20+ B cells/ml birth	29	-0.12
No CD24hiCD38low Memory of CD20+ B cells/ml birth	21	-0.05
% CD24hiCD38low Memory of CD20+ B cells birth	32	0.03
No CD24lowCD38low Naive of CD20+ B cells/ml birth	21	-0.30
% CD24lowCD38low Naive of CD20+ B cells birth	32	-0.01
No CD24hiCD38hi Transitional of CD20+ B cells/ml birth	21	-0.15
% CD24hiCD38hi Transitional of CD20+ B cells birth	32	-0.09
% CD27+ Memory of CD20+ B cells birth	32	-0.53
% IgM-CD27+ memory B cells birth	32	-0.48
% IgM+CD27+ memory B cells birth	32	-0.28
% IgM+CD27-naive B cells birth	32	-0.13
% IgD+ of IgM+CD27- naive B cells birth	32	-0.20
No of CD4 T cells/ml birth	31	-0.14
% CD31-CD45RA+ of CD4+ T cells birth	28	-0.16
% CD31+CD45RA+ of CD4+ T cells birth	28	-0.13
% CCR7+CD45RA+ of CD4+ T cells birth	28	-0.45
% CCR7+CD45RA- of CD4+ T cells birth	28	-0.24
% CCR7-CD45RA- of CD4+ T cells birth	28	-0.18
No of CD8+ T cells/ml birth	31	-0.00
% CCR7-CD45RA+ of CD8+ T cells birth	27	-0.55
% CCR7+CD45RA+ of CD8+ T cells birth	27	-0.27
% CCR7+CD45RA- of CD8+ T cells birth	27	-0.10
% CCR7-CD45RA- of CD8+ T cells birth	27	-0.18
% CD31-CD45RA+ of CD4+ T cells 48h	21	-0.17
% CD31+CD45RA+ of CD4+ T cells 48h	21	-0.33
% CD31-CD45RA+ of CD4+ T cells 1m	22	-0.05
% CD31+CD45RA+ of CD4+ T cells 1m	22	-0.19
% CCR7-CD45RA+ of CD4+ T cells 1m	21	-0.47
% CCR7+CD45RA+ of CD4+ T cells 1m	21	-0.04
% CCR7+CD45RA- of CD4+ T cells 1m	21	0.09
% CCR7-CD45RA- of CD4+ T cells 1m	21	-0.60
No of CD20+ B cells/ml 4m	24	-0.23
No of IgM-CD27+ of B cells 4m	21	-0.28
% IgM-CD27+ of B cells 4m	24	-0.17
No of IgM+CD27+ memory B cells 4m	21	-0.39
% IgM+CD27+ memory B cells 4m	24	-0.19
No of IgM+CD27- naive B cells 4m	21	-0.25
% IgM+CD27- naive B cells 4m	24	-0.20
No of IgD+ of IgM+CD27-naive B cells	21	-0.28
% IgD+ of IgM+CD27-naive B cells	24	-0.38
CD4 counts/ml 4m	24	-0.30
% CD31-CD45RA+ of CD4 T cells 4m	22	-0.37
% CD31+CD45RA+ of CD4+ T cells 4m	22	-0.50
% CCR7- CD45RA+ of CD4+ T cells 4m	25	0.15
% CCR7+CD45RA+ of CD4+ T cells 4m	25	-0.42
% CCR7+CD45RA- of CD4+ T cells 4m	25	0.29
% CCR7-CD45RA- of CD4+ T cells 4m	25	-0.54

No of CCR7-CD45RA+ of CD4+ T cells 4m	22	-0.31
No of CCR7+CD45RA+ of CD4+ T cells 4m	22	-0.42
No of CCR7+CD45RA- of CD4+ T cells 4m	22	-0.39
No of CCR7-CD45RA- of CD4+ T cells 4m	22	0.22
No of CD8+ T cells/ml 4m	22	-0.35
% CCR7-CD45RA+ CD8+ T cells 4m	22	-0.13
% CCR7+CD45RA+ CD8+ T cells 4m	22	-0.07
% CCR7+CD45RA- CD8+ T cells 4m	22	-0.10
% CCR7-CD45RA- CD8+ T cells 4m	22	0.02
No of CD20/ml 12m	26	-0.24
% IgM-CD27+ memory B cells at 12m	25	-0.12
% IgM+CD27+ memory B cells 12m	25	-0.51
% CD27+ memory B cells at 12m	25	-0.36
% IgM+CD27- naive B cells 12m	25	-0.28
% IgD+ of IgM+CD27- naive B cells 12m	24	0.07
% IgM-CD27- B cells 12m	25	-0.54
% CD24+CD38low memory B cells at 12m	23	0.18
% CD24hiCD38hi transitional B cells 12m	23	-0.26
% CD24low CD38low Naive B cells 12m	23	0.29
No of CD45/ml 12m	27	-0.41
No of CD4/ml 12m	27	-0.19
% CD31-CD45RA+ of CD4+ T cells 12m	26	0.01
% CD31+CD45RA+ of CD4+ T cells 12m	26	-0.32
% CD31+CD45RA- of CD4+ T cells 12m	26	-0.17
No of CD31-CD45RA+ of CD4+ T cells 12m	22	-0.33
No of CD31+CD45RA+ of CD4+ T cells 12m	22	-0.16
No of CD31+CD45RA- of CD4+ T cells 12m	22	-0.13
% CCR7-CD45RA+ of CD4+ T cells 12m	26	-0.20
% CCR7+CD45RA+ of CD4+ T cells 12m	26	0.10
% CCR7+CD45RA- of CD4+ T cells 12m	26	0.01
% CCR7-CD45RA- of CD4+ T cells 12m	26	0.11
No of CCR7-CD45RA+ of CD4+ T cells 12m	22	-0.42
No of CCR7+CD45RA+ of CD4+ T cells 12m	22	-0.35
No of CCR7+CD45RA- of CD4+ T cells 12m	22	-0.26
No of CCR7-CD45RA- of CD4+ T cells 12m	22	-0.61
No of CD8/ml 12m	27	-0.35
% CCR7-CD45RA+ of CD8+ T cells 12m	26	-0.14
% CCR7+CD45RA+ of CD8+ T cells 12m	26	-0.26
% CCR7+CD45RA- of CD8+ T cells 12m	26	-0.01
% CCR7-CD45RA- of CD8+ T cells 12m	26	-0.17
No of CCR7-CD45RA+ of CD8+ T cells 12m	22	-0.65
No of CCR7+CD45RA+ of CD8+ T cells 12m	22	-0.42
No of CCR7+CD45RA- of CD8+ T cells 12m	22	-0.59
No of CCR7-CD45RA- of CD8+ T cells 12m	22	-0.51

Table S5. Antibodies used in the flow cytometry analysis

Cellpopulation studied	Monoclonal antibody	Fluorochrome	Clone	Manufacture
TruCount (cell numbers)	anti-CD45<br>anti-CD4<br>anti-CD8<br>anti-CD20	FITC<br>PerCP<br>PE<br>APC	HI30<br>SK3<br>SK1<br>L27	BD Bioscience<br>BD Bioscience<br>BD Bioscience<br>BD Bioscience
Transitional B cells	anti-CD20<br>anti-CD24<br>anti-CD38<br>anti-CD5	PerCP<br>AF647<br>PE<br>FITC	L27<br>ML5<br>HB7<br>UCHT2	BD Bioscience<br>BD Bioscience<br>BD Bioscience<br>BD Bioscience
memory B cells	anti-CD20<br>anti-CD27<br>anti-IgD<br>anti-IgM	PerCP<br>FITC<br>PE<br>APC	L27<br>L128<br>IA6-2<br>G20-127	BD Bioscience<br>BD Bioscience<br>BD Bioscience<br>BD Bioscience
Recent thymic emigrants	anti-CD4<br>anti-CD31<br>anti-CD45RA<br>anti-CD127	PerCP<br>PE<br>APC<br>FITC	SK3<br>WM59<br>HI100<br>HIL-7R-M21	BD Bioscience<br>BD Bioscience<br>BD Bioscience<br>BD Bioscience
Naïve and memory T cells	anti-CD4<br>anti-CD8<br>anti-CD45RA<br>anti-CCR7<br>anti-CD44	PerCP<br>PerCP<br>APC<br>PE<br>FITC	SK3<br>SK1<br>HI100<br>G043H7<br>IM7	BD Bioscience<br>BD Bioscience<br>BD Bioscience<br>BioLegend<br>BioLegend

Abbreviations: APC, Allophycocyanin; PerCp, Peridinin Cholorophyll Protein Complex; FITC, fluorescein isothiocyanate; and PE, R-Phycoerythrin

Table S6. All features belonging to clusters that showed to associate with significant outcomes.

Cluster name	Features (mz@rt)
RNCC1064	596.4267@434.76<br>370.21301@434.88
HNCC249	315.25757@52.5<br>329.2338@52.74
RNCC1138	568.79736@32.94<br>570.83838@33.24
RNCC0424	738.91449@35.88<br>746.80267@35.88<br>838.73096@35.88<br>914.71008@35.88<br>932.81158@35.94
RPCC0009	274.87427@35.82<br>342.86136@35.82<br>648.80304@35.82<br>732.76703@35.82<br>1156.66455@35.82<br>1190.65955@35.82<br>1258.64954@35.82<br>410.84906@35.88<br>478.83691@35.88<br>546.82416@35.88<br>614.81171@35.88<br>766.76068@35.88<br>834.74805@35.88<br>868.73877@35.88<br>902.73535@35.88<br>970.7243@35.88<br>986.69952@35.88<br>1054.68591@35.88<br>1122.67236@35.88<br>630.78558@35.94<br>698.77319@35.94

Table S7. Evaluation of Peak Quality (Table and Extracted Ion Chromatograms).

feature	mode	chrom	pol	mz	rt (s)	analysis	r	p	comment on peak quality
HN112.9861@343.62	HN	HILIC	NEG	112,9861	343,62	KREC in umbilical cord blood	-0,31	0,06	1st peak in a cluster of peaks
HN329.2338@52.742	HN	HILIC	NEG	329,2338	52,742	Proportion of CD24low CD38low naive B cells of total B cells at 12 months	-0,74	0,001	good
HN92.05019@54.42	HN	HILIC	NEG	92,05019	54,42	Proportion of CD24low CD38low naive B cells of total B cells at 12 months	-0,61	0,002	ok
HP422.10547@368.64	HP	HILIC	POS	422,1055	368,64	Number of CCR7+ CD45RA- T cells at 4 months	-0,65	0,001	good
RN568.79736@32.942	RN	Reversed Phase	NEG	568,7974	32,942	Proportion of CD24low CD38low naive B cells of total B cells at 12 months	0,52	0,011	good
RN196.94266@36.36	RN	Reversed Phase	NEG	196,9427	36,36	Proportion of CD24low CD38low naive B cells of total B cells at 12 months	0,62	0,002	good
RN746.80267@35.882	RN	Reversed Phase	NEG	746,8027	35,882	Proportion of CD24low CD38low naive B cells of total B cells at 12 months	0,65	0,001	ok
RN596.4267@434.762	RN	Reversed Phase	NEG	596,4267	434,762	Proportion of CCR7+ CD45RA- T cells of CD4+ T cells at 4 months	0,65	0,001	good
RN245.07942@348.66	RN	Reversed Phase	NEG	245,0794	348,66	Proportion of CCR7+ CD45RA- T cells of CD4+ T cells at 4 months	0,74	0,001	ok
RN381.16165@455.22	RN	Reversed Phase	NEG	381,1617	455,22	Proportion of CCR7+ CD45RA- T cells of CD4+ T cells at 4 months	0,78	0,001	good
RN145.03938@121.62	RN	Reversed Phase	NEG	145,0394	121,62	Proportion of CCR7+ CD45RA- T cells of CD4+ T cells at 4 months	0,67	0,001	ok
RN417.17871@443.58	RN	Reversed Phase	NEG	417,1787	443,58	Proportion of CCR7+ CD45RA- T cells of CD4+ T cells at 4 months	0,69	0,001	ok
RP285.27487@418.98	RP	Reversed Phase	POS	285,2749	418,98	KREC in umbilical cord blood	-0,55	0,001	ok
RP579.18646@327.72	RP	Reversed Phase	POS	579,1865	327,72	KREC in umbilical cord blood	-0,46	0,005	ok
RP279.07724@362.7	RP	Reversed Phase	POS	279,0772	362,7	KREC in umbilical cord blood	0,6	0,001	good
RP511.27478@207.3	RP	Reversed Phase	POS	511,2748	207,3	KREC in umbilical cord blood	-0,27	0,1	small, only present in some samples
RP78.03462@76.56	RP	Reversed Phase	POS	78,03462	76,56	Proportion of CD24low CD38low naive B cells of total B cells at 12 months	0,71	0,001	ok
RP379.13776@185.76	RP	Reversed Phase	POS	379,1378	185,76	Proportion of CD24low CD38low naive B cells of total B cells at 12 months	-0,48	0,021	ok
RP175.15396@325.68	RP	Reversed Phase	POS	175,154	325,68	Proportion of CD24low CD38low naive B cells of total B cells at 12 months	-0,47	0,024	ok
RP319.14124@170.4	RP	Reversed Phase	POS	319,1412	170,4	Proportion of CD24low CD38low naive B cells of total B cells at 12 months	-0,54	0,008	good
RP274.87427@35.822	RP	Reversed Phase	POS	274,8743	35,822	Proportion of CD24low CD38low naive B cells of total B cells at 12 months	0,63	0,001	good
RP332.25632@272.64	RP	Reversed Phase	POS	332,2563	272,64	Number of CCR7+ CD45RA- T cells at 4 months	0,63	0,001	good
RP386.29367@329.64	RP	Reversed Phase	POS	386,2937	329,64	Number of CCR7+ CD45RA- T cells at 4 months	0,69	0,001	good
RP358.26218@296.10	RP	Reversed Phase	POS	358,2622	296,1	Number of CCR7+ CD45RA- T cells at 4 months	0,68	0,001	ok
RP464.33435@365.28	RP	Reversed Phase	POS	464,3344	365,28	Number of CCR7+ CD45RA- T cells at 4 months	0,76	0,001	ok, only present in some samples, in cluster of peaks
RP228.08873@116.88	RP	Reversed Phase	POS	228,0887	116,88	Number of CCR7+ CD45RA- T cells at 4 months	0,59	0,002	small, only present in some samples
RP442.37125@366.12	RP	Reversed Phase	POS	442,3713	366,12	Number of CCR7+ CD45RA- T cells at 4 months	0,69	0,001	good
RP360.28867@312.00	RP	Reversed Phase	POS	360,2887	312	Number of CCR7+ CD45RA- T cells at 4 months	0,64	0,001	good
RP273.10336@39.84	RP	Reversed Phase	POS	273,1034	39,84	Proportion of CCR7+ CD45RA- T cells of CD4+ T cells at 4 months	0,61	0,002	good

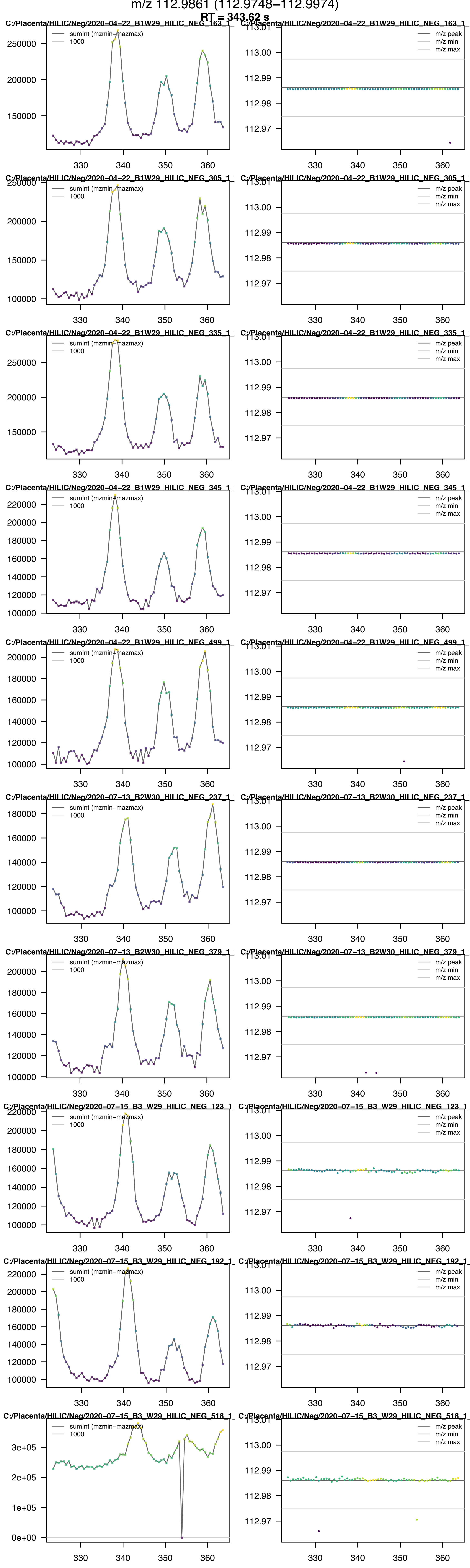
m/z 112.9861 (112.9748–112.9974)
RT = 343.62 s
C:/Placenta/HILIC/Neg/2020-04-22_B1W29_HILIC_NEG_163_1
C:/Placenta/HILIC/Neg/2020-04-22_B1W29_HILIC_NEG_305_1
C:/Placenta/HILIC/Neg/2020-04-22_B1W29_HILIC_NEG_335_1
C:/Placenta/HILIC/Neg/2020-04-22_B1W29_HILIC_NEG_345_1
C:/Placenta/HILIC/Neg/2020-04-22_B1W29_HILIC_NEG_499_1
C:/Placenta/HILIC/Neg/2020-07-13_B2W30_HILIC_NEG_237_1
C:/Placenta/HILIC/Neg/2020-07-13_B2W30_HILIC_NEG_379_1
C:/Placenta/HILIC/Neg/2020-07-15_B3_W29_HILIC_NEG_123_1
C:/Placenta/HILIC/Neg/2020-07-15_B3_W29_HILIC_NEG_192_1
C:/Placenta/HILIC/Neg/2020-07-15_B3_W29_HILIC_NEG_518_1
sumInt (mzmin–mazmax)
1000
m/z peak
m/z min
m/z max

m/z 329.2338 (329.20088−329.26672)

RT = 52.742 s

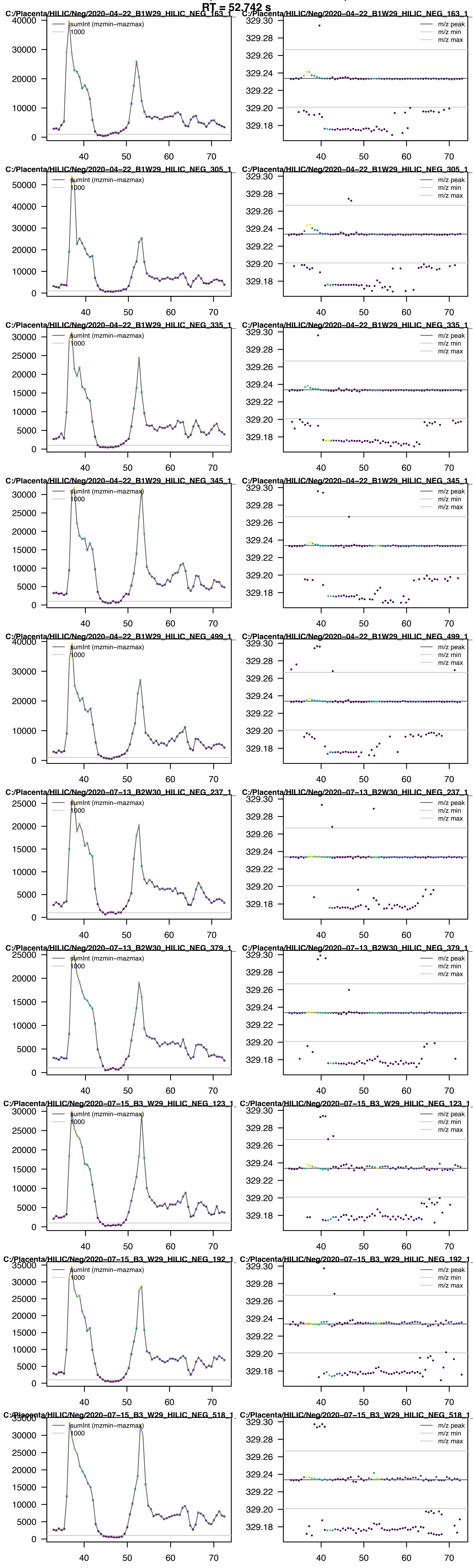

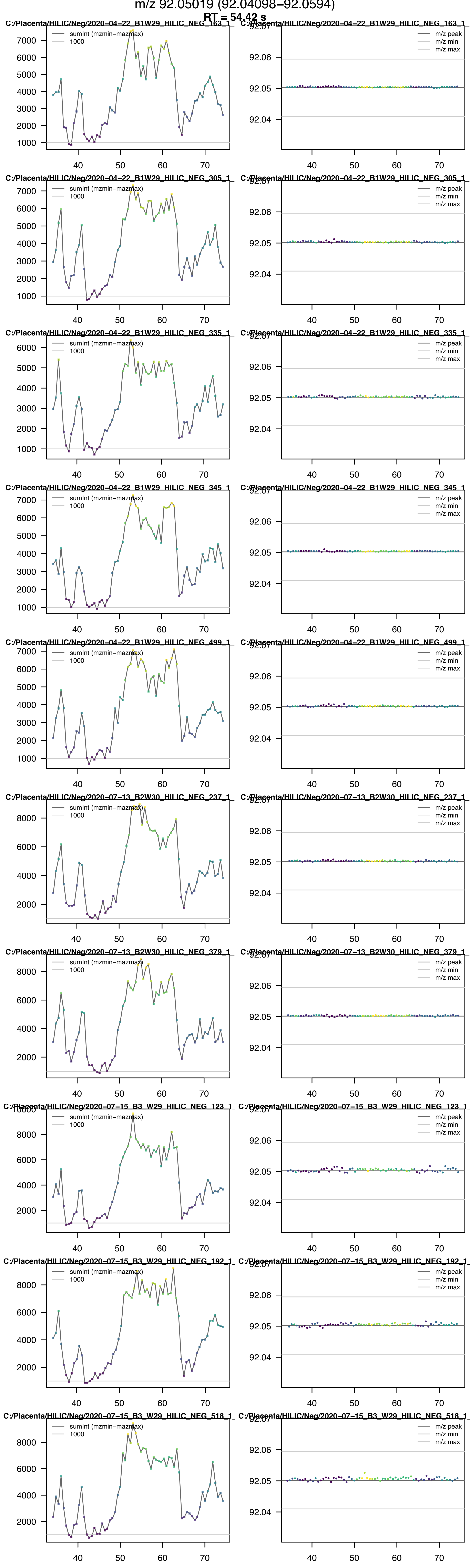
m/z 92.05019 (92.04098–92.0594)
RT = 54.42 s
C:/Placenta/HILIC/Neg/2020–04–22_B1W29_HILIC_NEG_163_1
C:/Placenta/HILIC/Neg/2020–04–22_B1W29_HILIC_NEG_305_1
C:/Placenta/HILIC/Neg/2020–04–22_B1W29_HILIC_NEG_335_1
C:/Placenta/HILIC/Neg/2020–04–22_B1W29_HILIC_NEG_345_1
C:/Placenta/HILIC/Neg/2020–04–22_B1W29_HILIC_NEG_499_1
C:/Placenta/HILIC/Neg/2020–07–13_B2W30_HILIC_NEG_237_1
C:/Placenta/HILIC/Neg/2020–07–13_B2W30_HILIC_NEG_379_1
C:/Placenta/HILIC/Neg/2020–07–15_B3_W29_HILIC_NEG_123_1
C:/Placenta/HILIC/Neg/2020–07–15_B3_W29_HILIC_NEG_192_1
C:/Placenta/HILIC/Neg/2020–07–15_B3_W29_HILIC_NEG_518_1
sumInt (mzmin–mazmax)
1000
m/z peak
m/z min
m/z max

m/z 422.10547 (422.06326–422.14768)
RT = 368.64 s

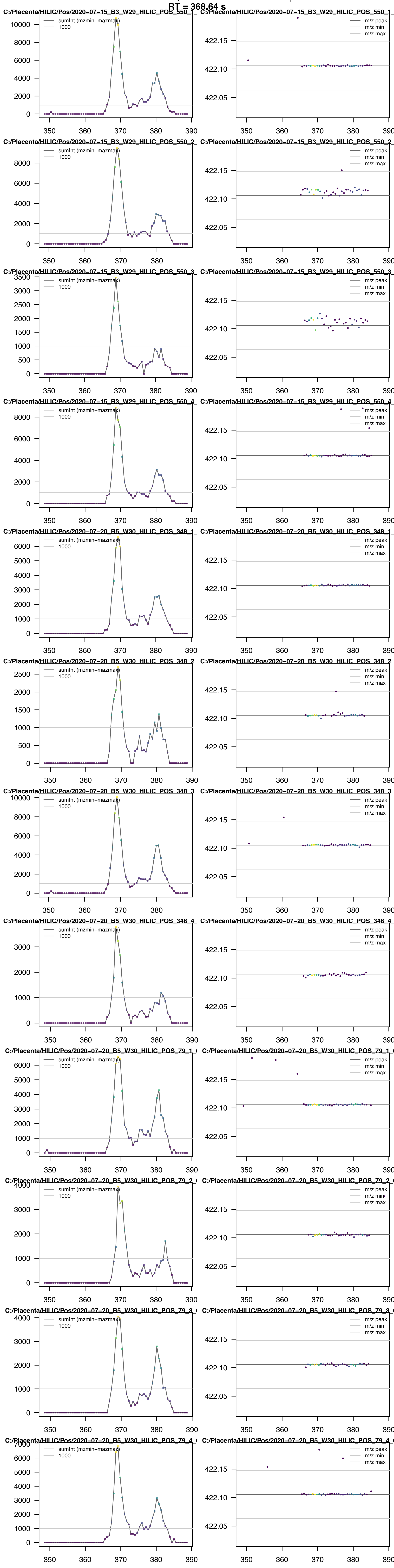

m/z 568.79736 (568.74048–568.85424)
RT = 32.942 s

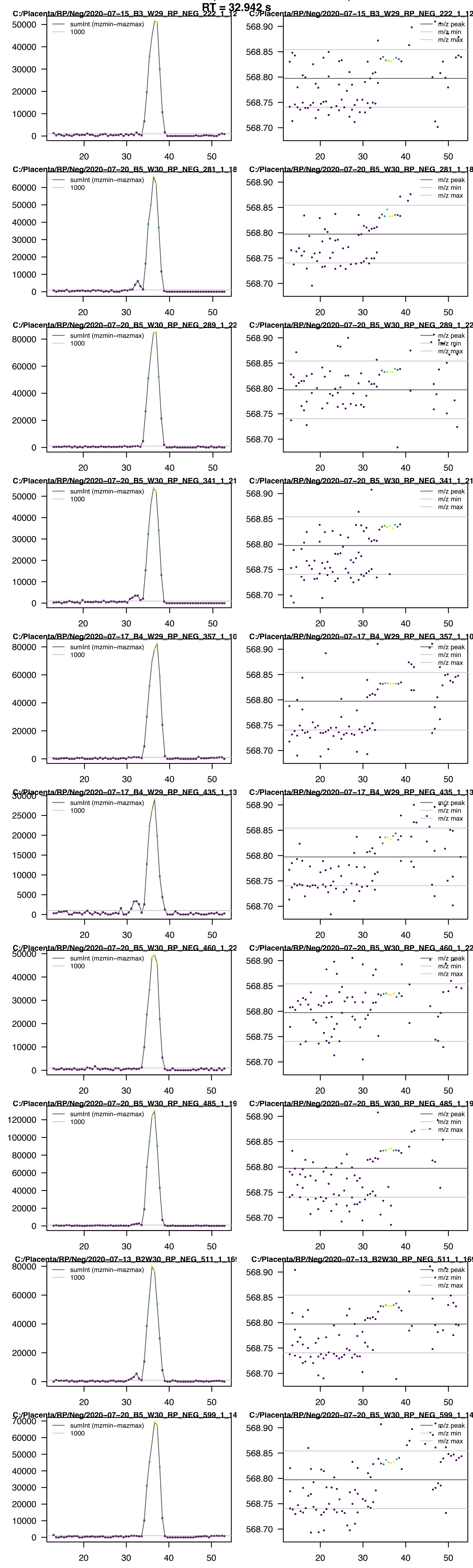

m/z 196.94266 (196.92297–196.96235)
RT = 36.36 s

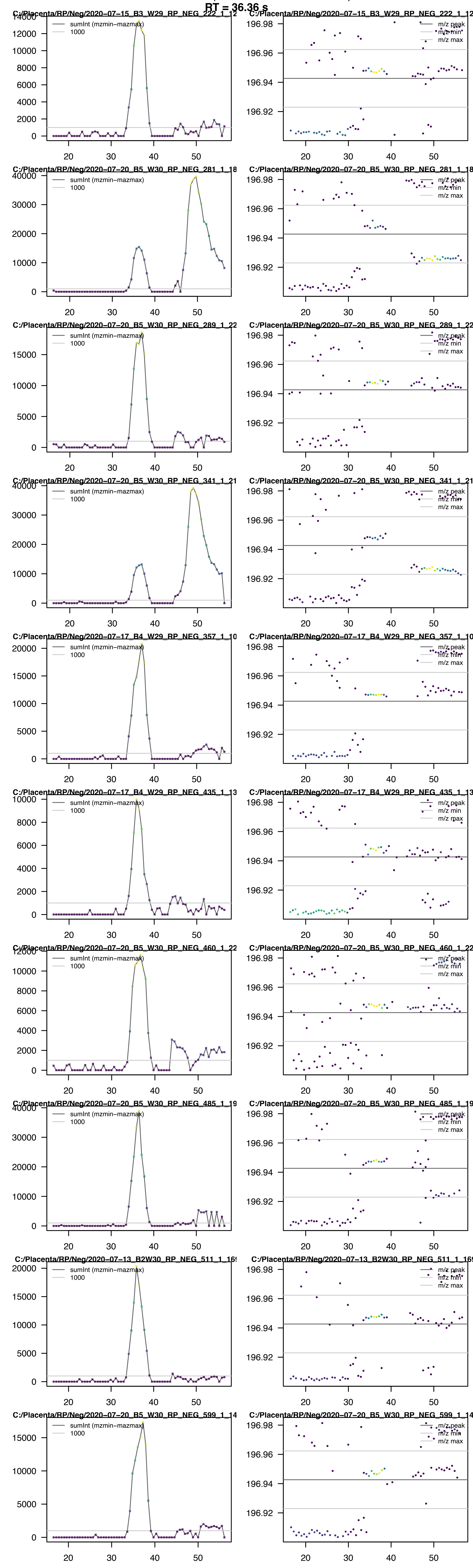

m/z 746.80267 (746.72799–746.87735)
RT = 35.882 s

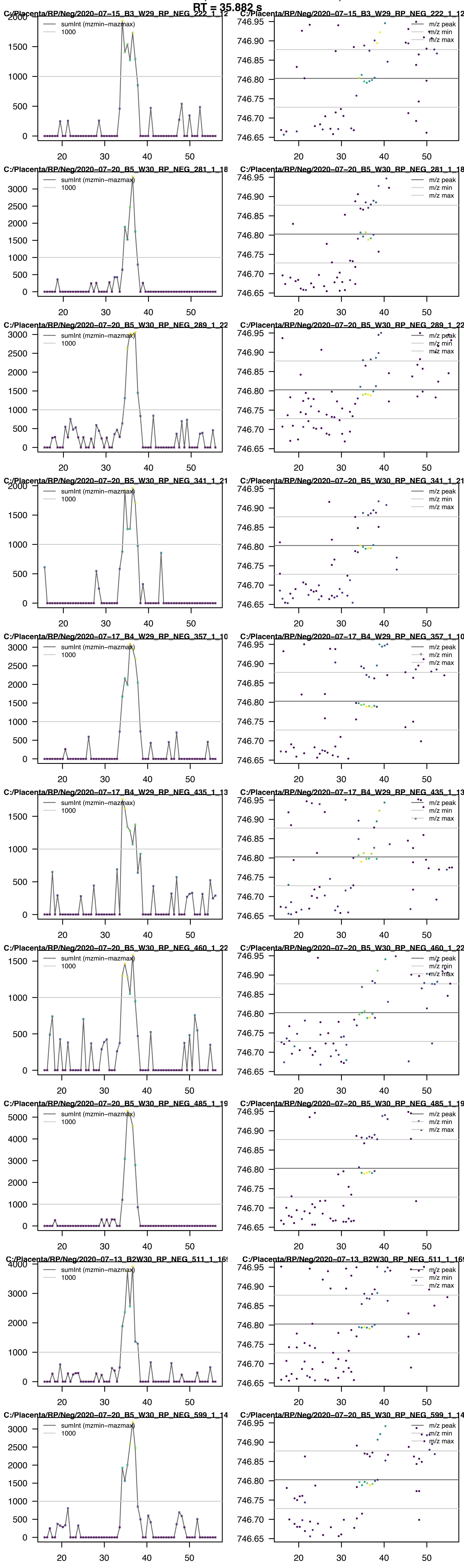

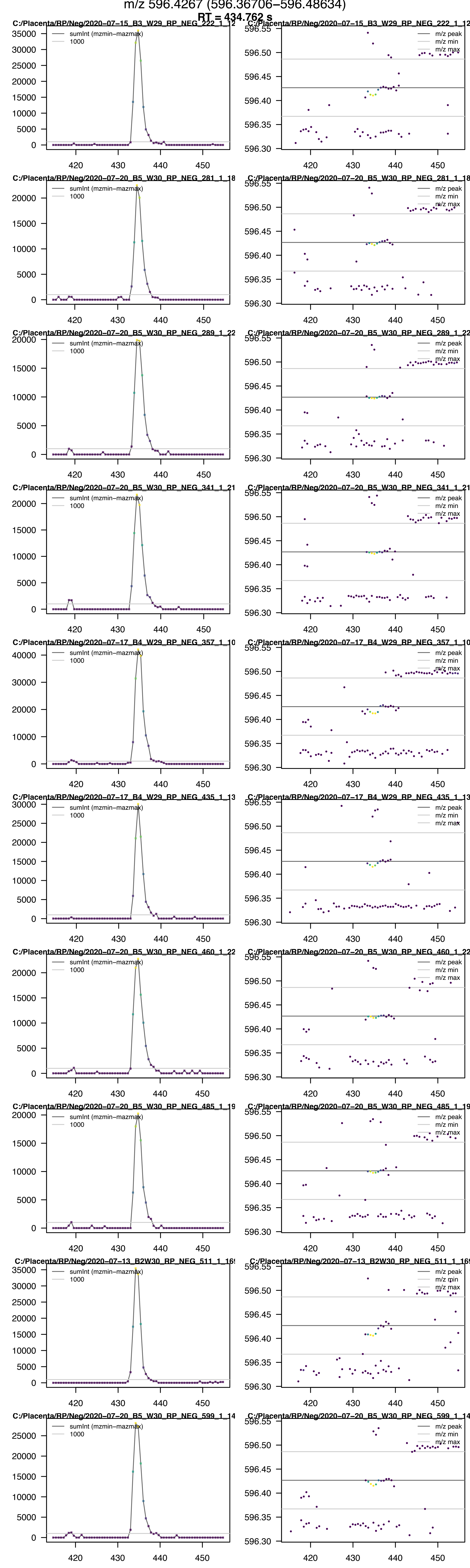
m/z 596.4267 (596.36706–596.48634)
RT = 434.762 s
C:/Placenta/RP/Neg/2020-07-15_B3_W29_RP_NEG_222_1_12
C:/Placenta/RP/Neg/2020-07-20_B5_W30_RP_NEG_281_1_18
C:/Placenta/RP/Neg/2020-07-20_B5_W30_RP_NEG_289_1_22
C:/Placenta/RP/Neg/2020-07-20_B5_W30_RP_NEG_341_1_21
C:/Placenta/RP/Neg/2020-07-17_B4_W29_RP_NEG_357_1_10
C:/Placenta/RP/Neg/2020-07-17_B4_W29_RP_NEG_435_1_13
C:/Placenta/RP/Neg/2020-07-20_B5_W30_RP_NEG_460_1_22
C:/Placenta/RP/Neg/2020-07-20_B5_W30_RP_NEG_485_1_19
C:/Placenta/RP/Neg/2020-07-13_B2W30_RP_NEG_511_1_16
C:/Placenta/RP/Neg/2020-07-20_B5_W30_RP_NEG_599_1_14
sumInt (mzmin–mazmax)
1000
m/z peak
m/z min
m/z max

m/z 245.07942 (245.05491–245.10393)
RT = 348.66 s

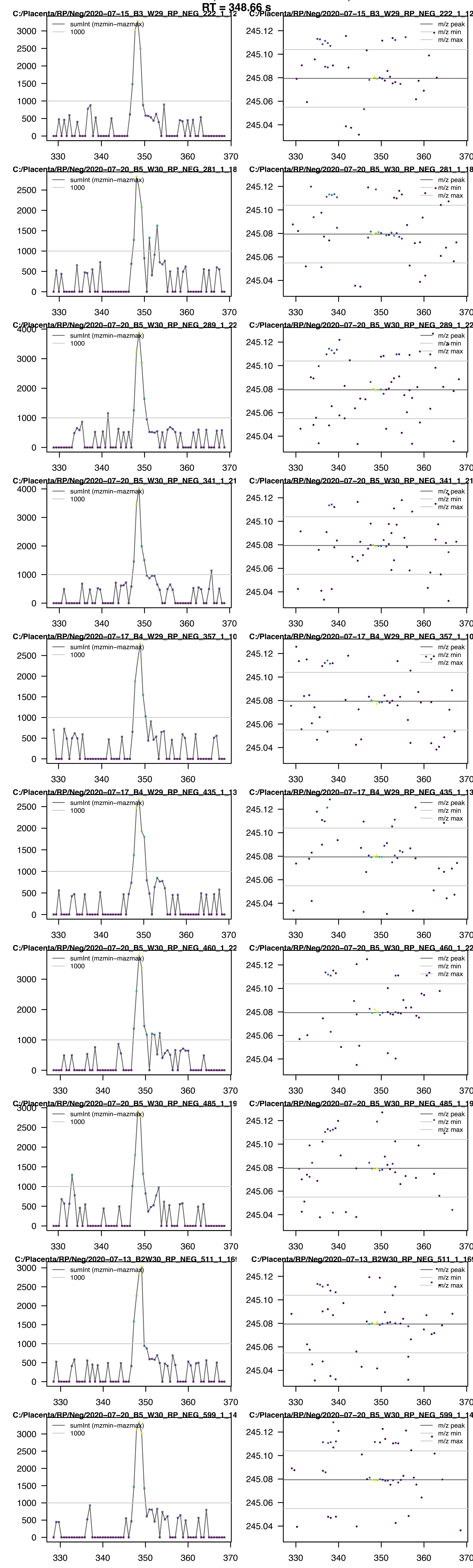

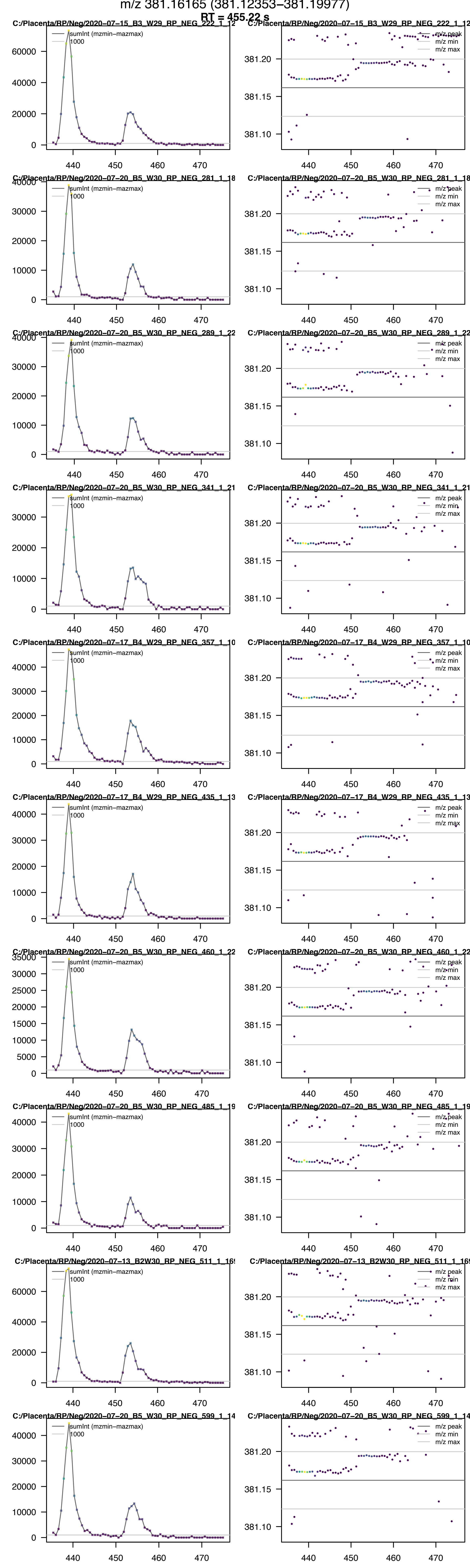

m/z 381.16165 (381.12353–381.19977)
RT = 455.22 s
C:/Placenta/RP/Neg/2020–07–15_B3_W29_RP_NEG_222_1_12
C:/Placenta/RP/Neg/2020–07–20_B5_W30_RP_NEG_281_1_18
C:/Placenta/RP/Neg/2020–07–20_B5_W30_RP_NEG_289_1_22
C:/Placenta/RP/Neg/2020–07–20_B5_W30_RP_NEG_341_1_21
C:/Placenta/RP/Neg/2020–07–17_B4_W29_RP_NEG_357_1_10
C:/Placenta/RP/Neg/2020–07–17_B4_W29_RP_NEG_435_1_13
C:/Placenta/RP/Neg/2020–07–20_B5_W30_RP_NEG_460_1_22
C:/Placenta/RP/Neg/2020–07–20_B5_W30_RP_NEG_485_1_19
C:/Placenta/RP/Neg/2020–07–13_B2W30_RP_NEG_511_1_16
C:/Placenta/RP/Neg/2020–07–20_B5_W30_RP_NEG_599_1_14
sumInt (mzmin–mazmax)
1000
m/z peak
m/z min
m/z max

m/z 145.03938 (145.02488–145.05388)
RT = 121.62 s

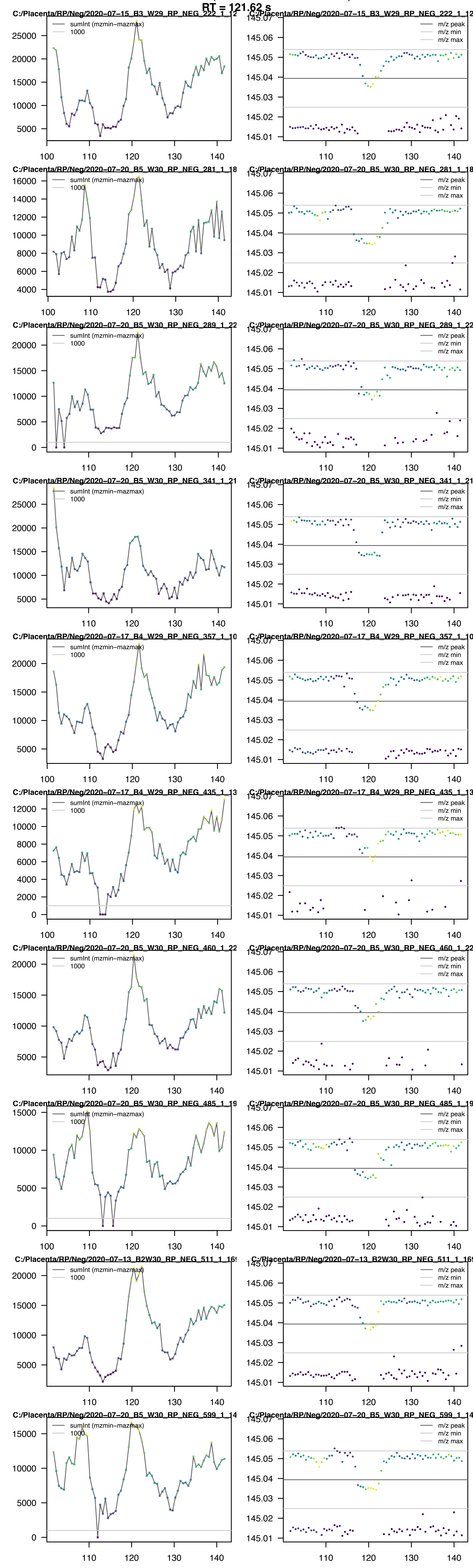

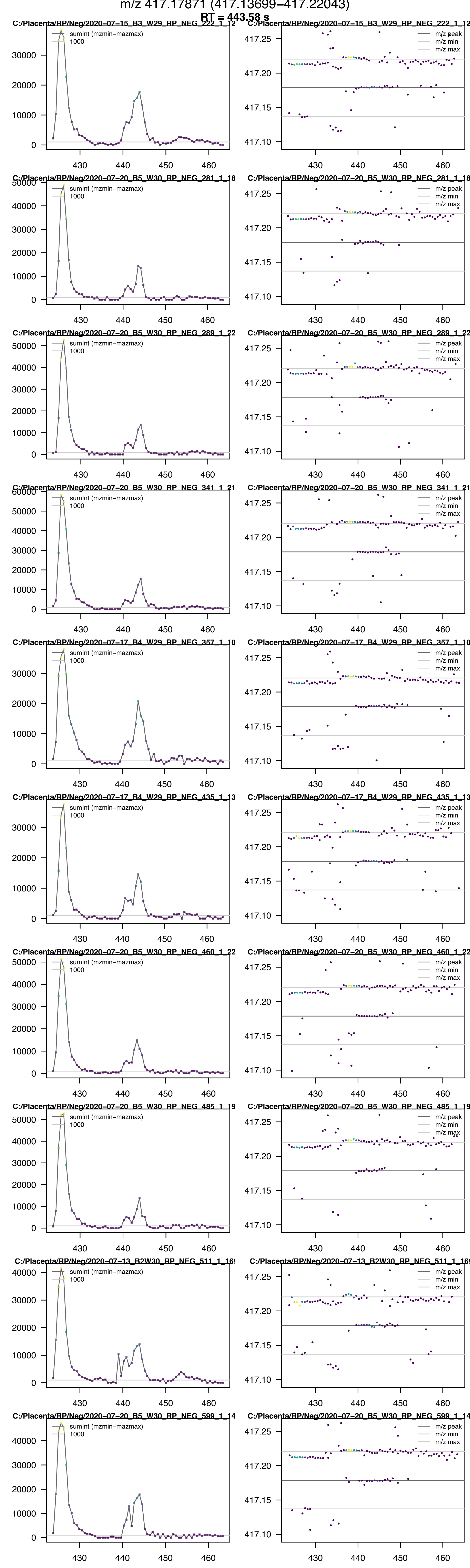

m/z 417.17871 (417.13699–417.22043)
RT = 443.58 s
C:/Placenta/RP/Neg/2020–07–15_B3_W29_RP_NEG_222_1_12
C:/Placenta/RP/Neg/2020–07–20_B5_W30_RP_NEG_281_1_18
C:/Placenta/RP/Neg/2020–07–20_B5_W30_RP_NEG_289_1_22
C:/Placenta/RP/Neg/2020–07–20_B5_W30_RP_NEG_341_1_21
C:/Placenta/RP/Neg/2020–07–17_B4_W29_RP_NEG_357_1_10
C:/Placenta/RP/Neg/2020–07–17_B4_W29_RP_NEG_435_1_13
C:/Placenta/RP/Neg/2020–07–20_B5_W30_RP_NEG_460_1_22
C:/Placenta/RP/Neg/2020–07–20_B5_W30_RP_NEG_485_1_19
C:/Placenta/RP/Neg/2020–07–13_B2W30_RP_NEG_511_1_169
C:/Placenta/RP/Neg/2020–07–20_B5_W30_RP_NEG_599_1_14
sumInt (mzmin–mazmax)
1000
m/z peak
m/z min
m/z max

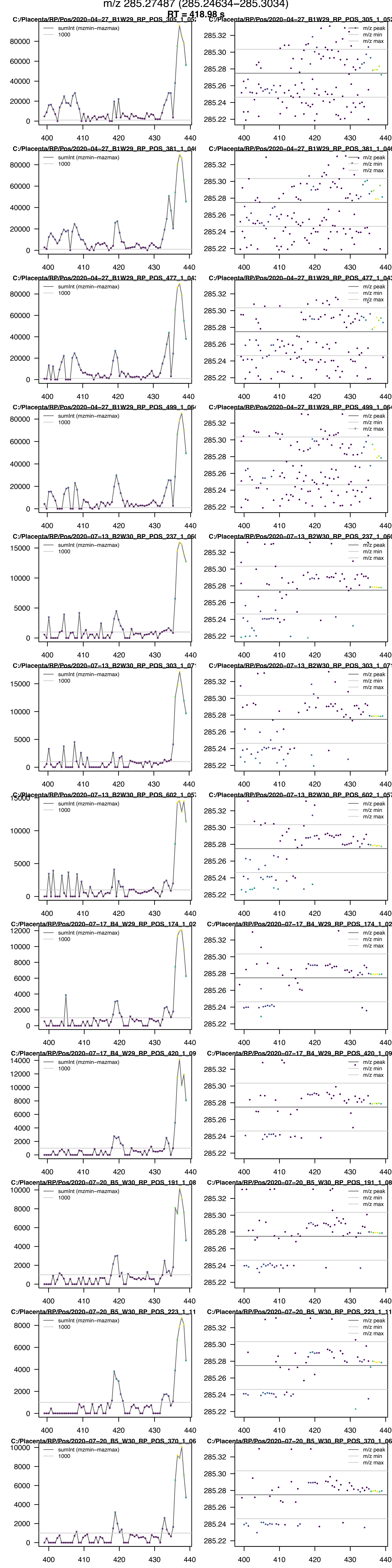
m/z 285.27487 (285.24634–285.3034)
RT = 418.98 s
C:/Placenta/RP/Pos/2020–04–27_B1W29_RP_POS_305_1_05
C:/Placenta/RP/Pos/2020–04–27_B1W29_RP_POS_381_1_04
C:/Placenta/RP/Pos/2020–04–27_B1W29_RP_POS_477_1_04
C:/Placenta/RP/Pos/2020–04–27_B1W29_RP_POS_499_1_06
C:/Placenta/RP/Pos/2020–07–13_B2W30_RP_POS_237_1_06
C:/Placenta/RP/Pos/2020–07–13_B2W30_RP_POS_303_1_07
C:/Placenta/RP/Pos/2020–07–13_B2W30_RP_POS_602_1_05
C:/Placenta/RP/Pos/2020–07–17_B4_W29_RP_POS_174_1_02
C:/Placenta/RP/Pos/2020–07–17_B4_W29_RP_POS_420_1_09
C:/Placenta/RP/Pos/2020–07–20_B5_W30_RP_POS_191_1_08
C:/Placenta/RP/Pos/2020–07–20_B5_W30_RP_POS_223_1_11
C:/Placenta/RP/Pos/2020–07–20_B5_W30_RP_POS_370_1_06
sumInt (mzmin–mazmax)
1000
m/z peak
m/z min
m/z max

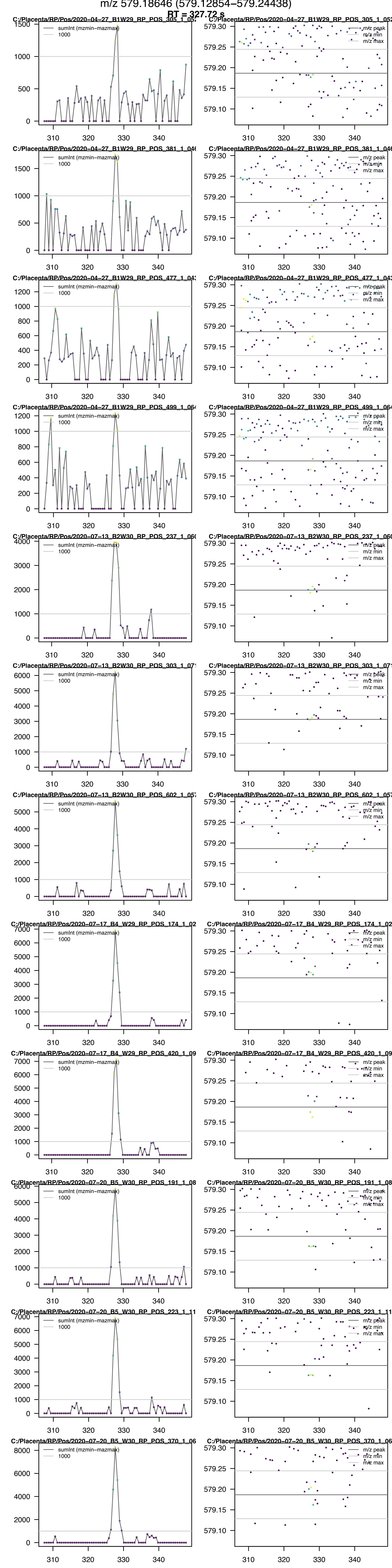
m/z 579.18646 (579.12854–579.24438)
RT = 327.72 s
C:/Placenta/RP/Pos/2020–04–27_B1W29_RP_POS_305_1_05
C:/Placenta/RP/Pos/2020–04–27_B1W29_RP_POS_381_1_04
C:/Placenta/RP/Pos/2020–04–27_B1W29_RP_POS_477_1_04
C:/Placenta/RP/Pos/2020–04–27_B1W29_RP_POS_499_1_06
C:/Placenta/RP/Pos/2020–07–13_B2W30_RP_POS_237_1_06
C:/Placenta/RP/Pos/2020–07–13_B2W30_RP_POS_303_1_07
C:/Placenta/RP/Pos/2020–07–13_B2W30_RP_POS_602_1_05
C:/Placenta/RP/Pos/2020–07–17_B4_W29_RP_POS_174_1_02
C:/Placenta/RP/Pos/2020–07–17_B4_W29_RP_POS_420_1_09
C:/Placenta/RP/Pos/2020–07–20_B5_W30_RP_POS_191_1_08
C:/Placenta/RP/Pos/2020–07–20_B5_W30_RP_POS_223_1_11
C:/Placenta/RP/Pos/2020–07–20_B5_W30_RP_POS_370_1_06
sumInt (mzmin–mazmax)
1000
m/z peak
m/z min
m/z max

m/z 279.07724 (279.04933–279.10515)
RT = 362.7 s

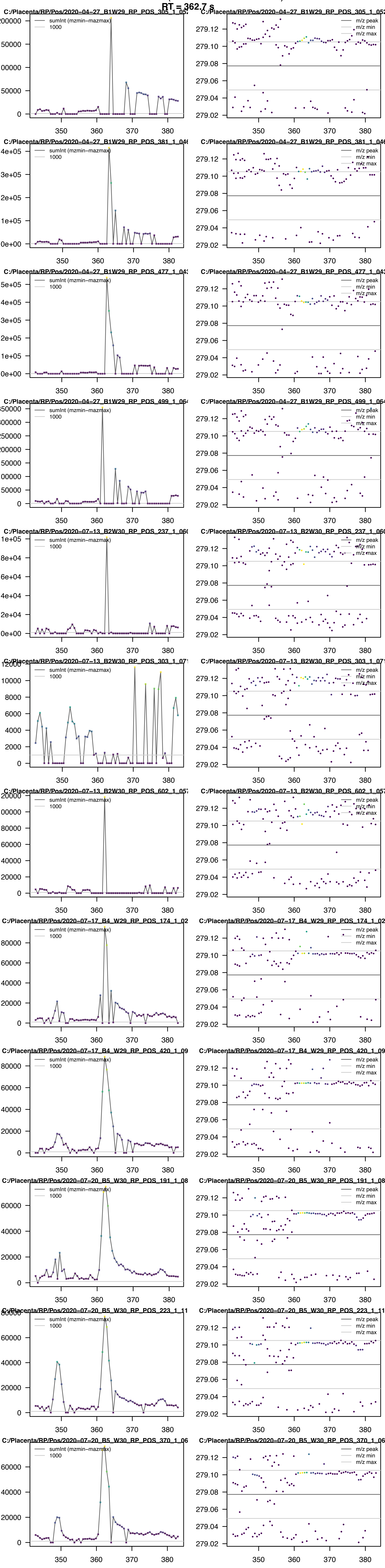

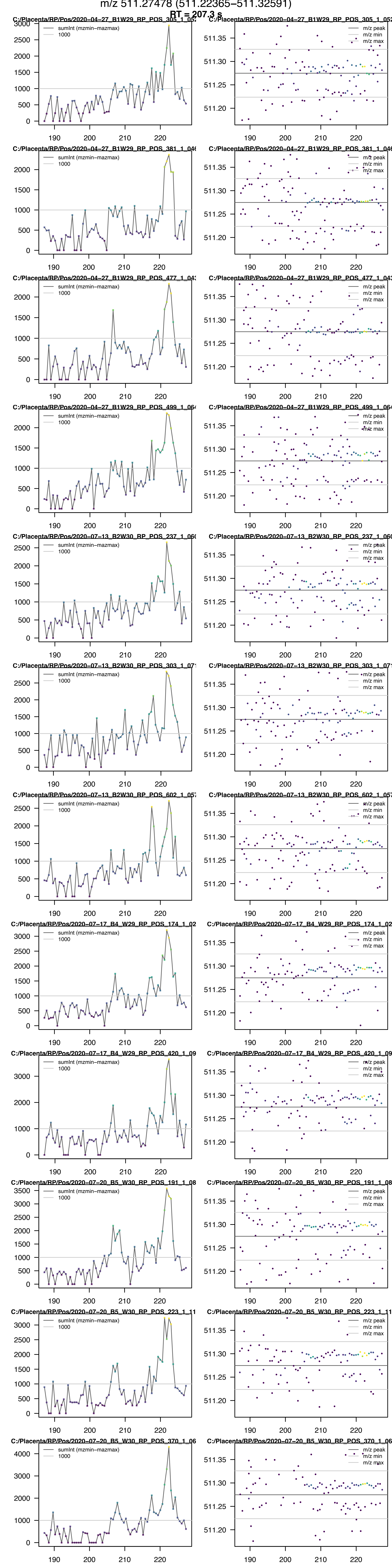
m/z 511.27478 (511.22365–511.32591)
RT = 207.3 s
C:/Placenta/RP/Pos/2020-04-27_B1W29_RP_POS_305_1_05
C:/Placenta/RP/Pos/2020-04-27_B1W29_RP_POS_381_1_04
C:/Placenta/RP/Pos/2020-04-27_B1W29_RP_POS_477_1_04
C:/Placenta/RP/Pos/2020-04-27_B1W29_RP_POS_499_1_06
C:/Placenta/RP/Pos/2020-07-13_B2W30_RP_POS_237_1_06
C:/Placenta/RP/Pos/2020-07-13_B2W30_RP_POS_303_1_07
C:/Placenta/RP/Pos/2020-07-13_B2W30_RP_POS_602_1_05
C:/Placenta/RP/Pos/2020-07-17_B4_W29_RP_POS_174_1_02
C:/Placenta/RP/Pos/2020-07-17_B4_W29_RP_POS_420_1_09
C:/Placenta/RP/Pos/2020-07-20_B5_W30_RP_POS_191_1_08
C:/Placenta/RP/Pos/2020-07-20_B5_W30_RP_POS_223_1_11
C:/Placenta/RP/Pos/2020-07-20_B5_W30_RP_POS_370_1_06
sumInt (mzmin–mazmax)
1000
m/z peak
m/z min
m/z max
190
200
210
220

m/z 78.03462 (78.02682–78.04242)
RT = 76.56 s

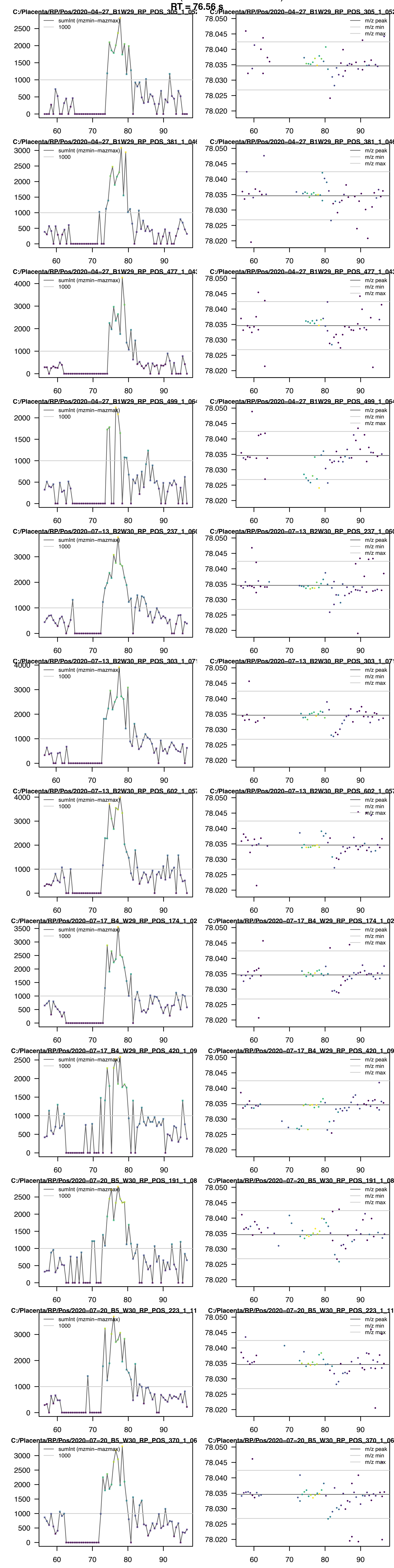

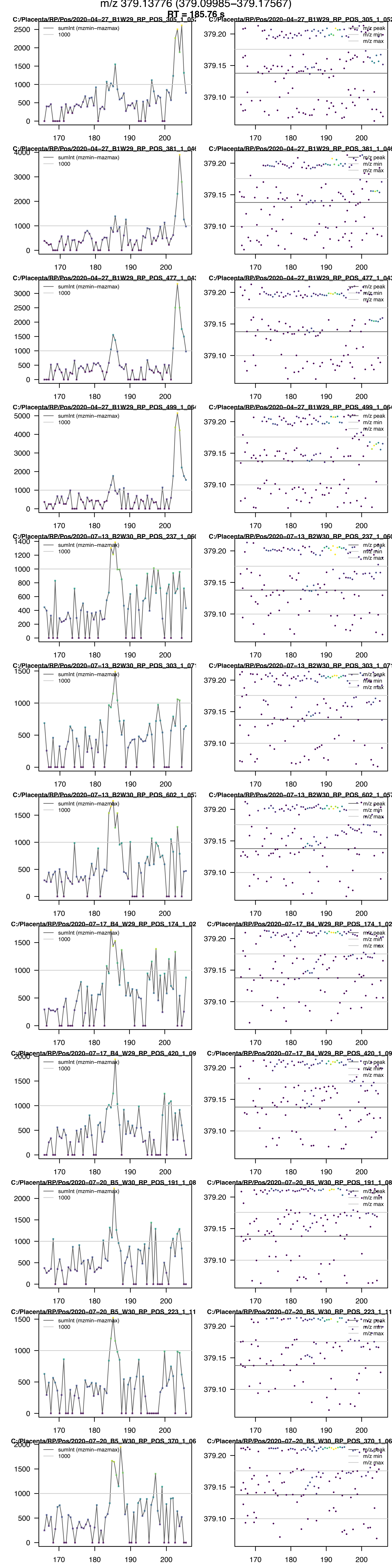
m/z 379.13776 (379.09985–379.17567)
RT = 185.76 s
C:/Placenta/RP/Pos/2020–04–27_B1W29_RP_POS_305_1_05
C:/Placenta/RP/Pos/2020–04–27_B1W29_RP_POS_381_1_04
C:/Placenta/RP/Pos/2020–04–27_B1W29_RP_POS_477_1_04
C:/Placenta/RP/Pos/2020–04–27_B1W29_RP_POS_499_1_06
C:/Placenta/RP/Pos/2020–07–13_B2W30_RP_POS_237_1_06
C:/Placenta/RP/Pos/2020–07–13_B2W30_RP_POS_303_1_07
C:/Placenta/RP/Pos/2020–07–13_B2W30_RP_POS_602_1_05
C:/Placenta/RP/Pos/2020–07–17_B4_W29_RP_POS_174_1_02
C:/Placenta/RP/Pos/2020–07–17_B4_W29_RP_POS_420_1_09
C:/Placenta/RP/Pos/2020–07–20_B5_W30_RP_POS_191_1_08
C:/Placenta/RP/Pos/2020–07–20_B5_W30_RP_POS_223_1_11
C:/Placenta/RP/Pos/2020–07–20_B5_W30_RP_POS_370_1_06
sumInt (mzmin–mazmax)
1000
m/z peak
m/z min
m/z max

m/z 175.15396 (175.13644–175.17148)
RT = 325.68 s

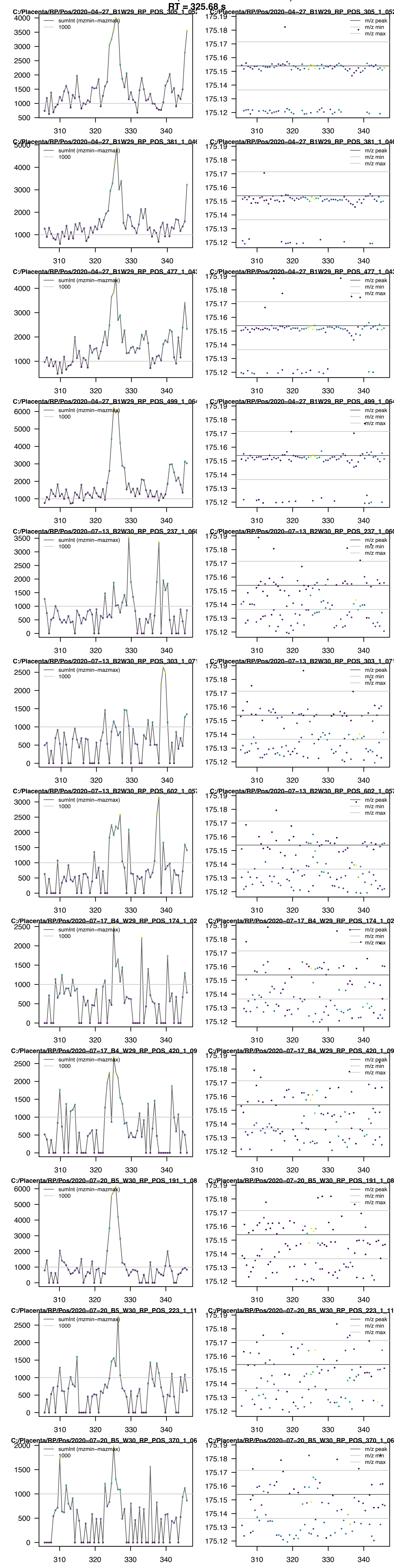

m/z 319.14124 (319.10933–319.17315)

RT = 170.4 s

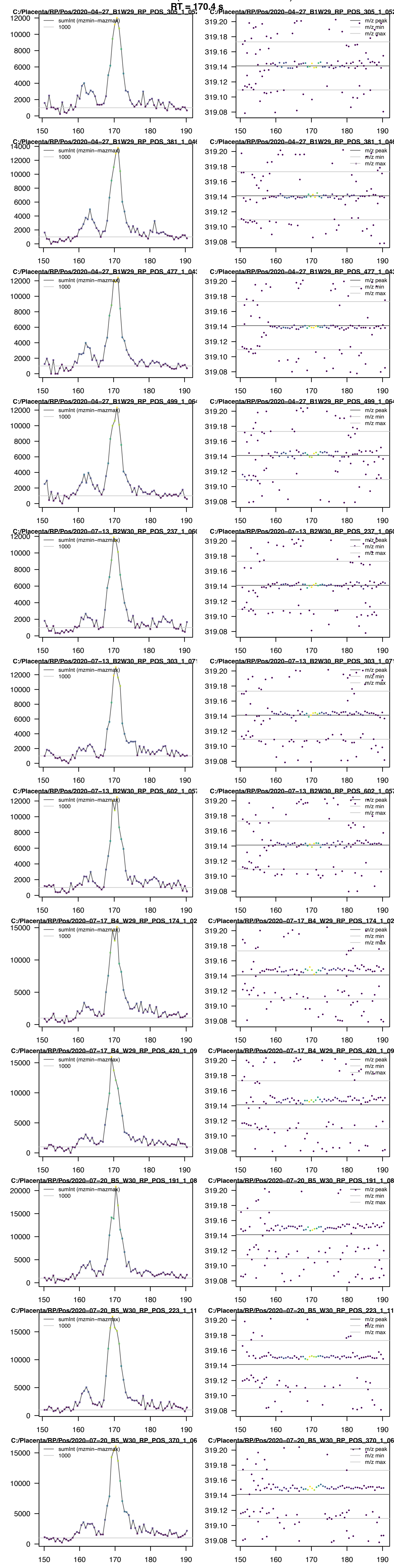

m/z 274.87427 (274.84678–274.90176)
RT = 35.822 s

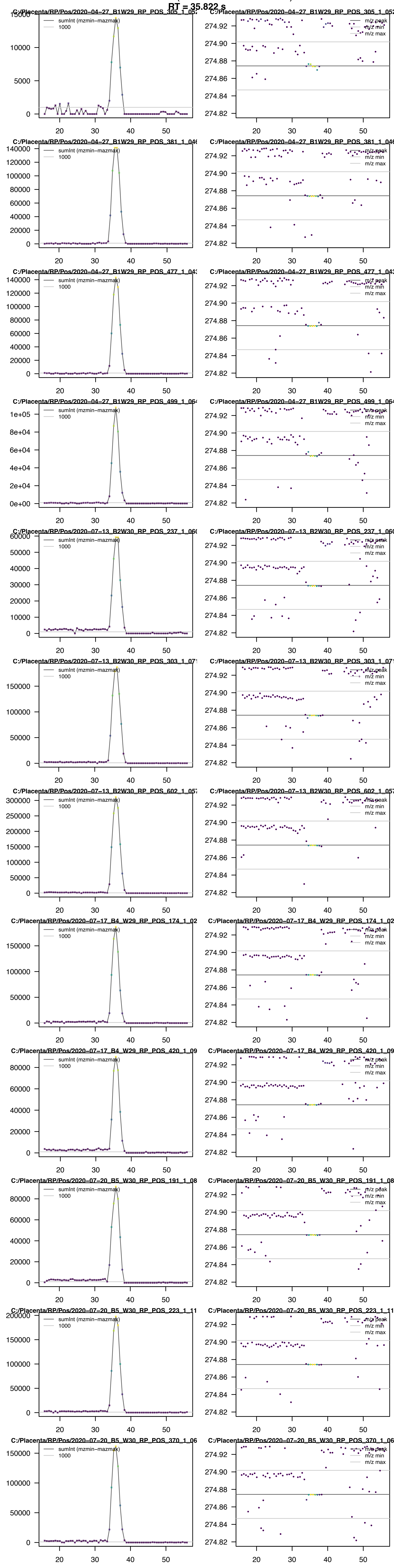

m/z 332.25632 (332.22309–332.28955)
RT = 272.64 s

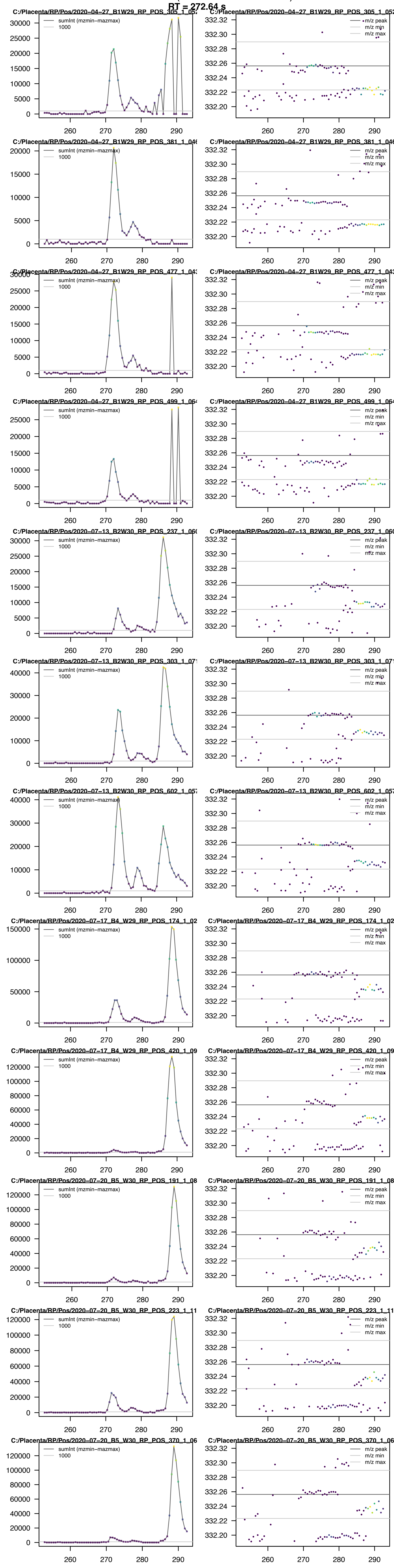

m/z 386.29367 (386.25504–386.3323)
RT = 329.64 s

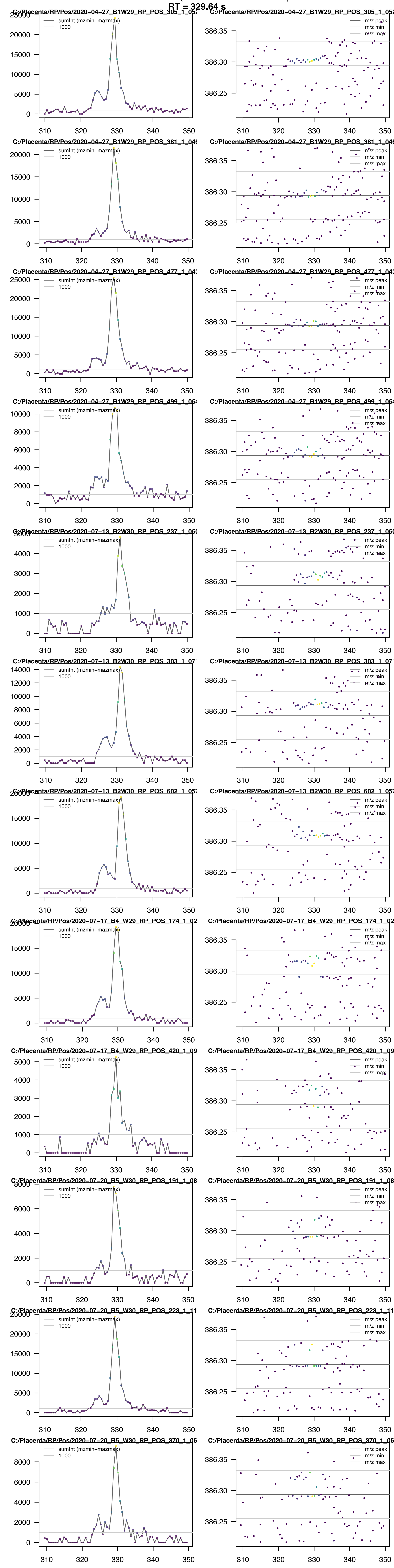

m/z 358.26218 (358.22635–358.29801)
RT = 296.1 s

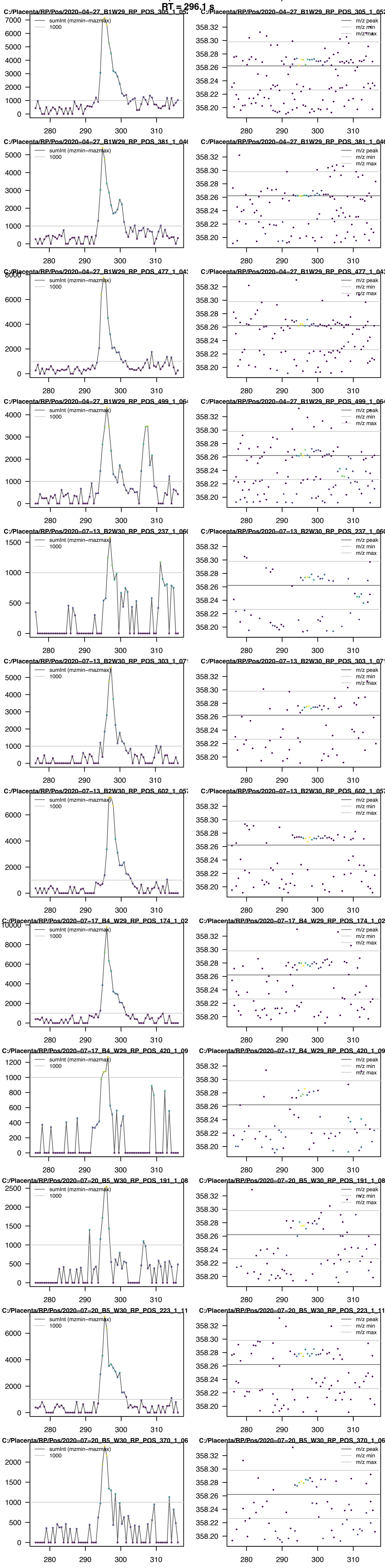

m/z 464.33435 (464.28792–464.38078)

RT = 365.28 s

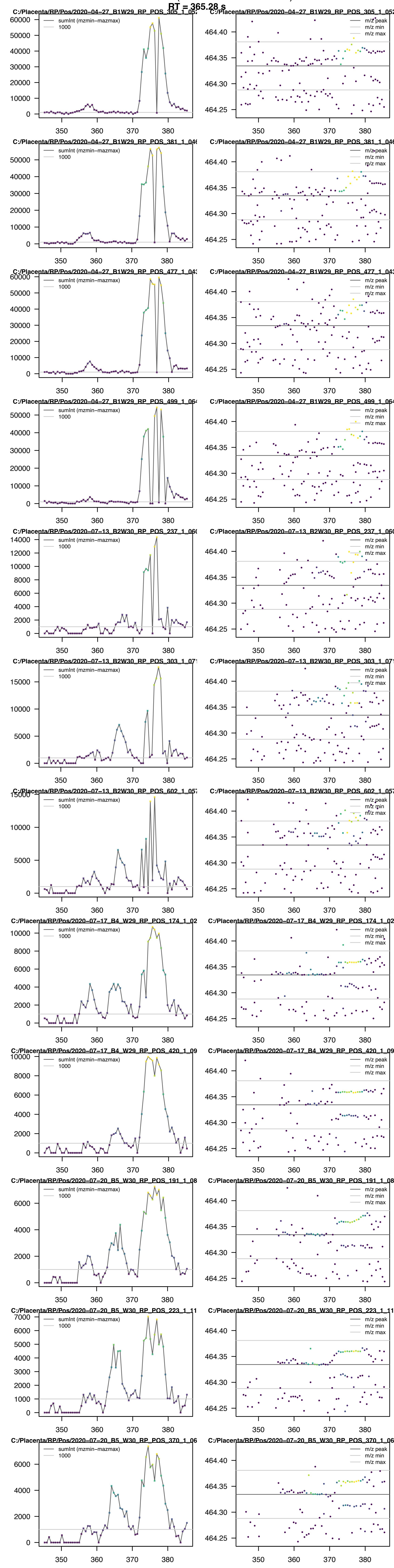

m/z 228.08873 (228.06592–228.11154)
RT = 116.88 s

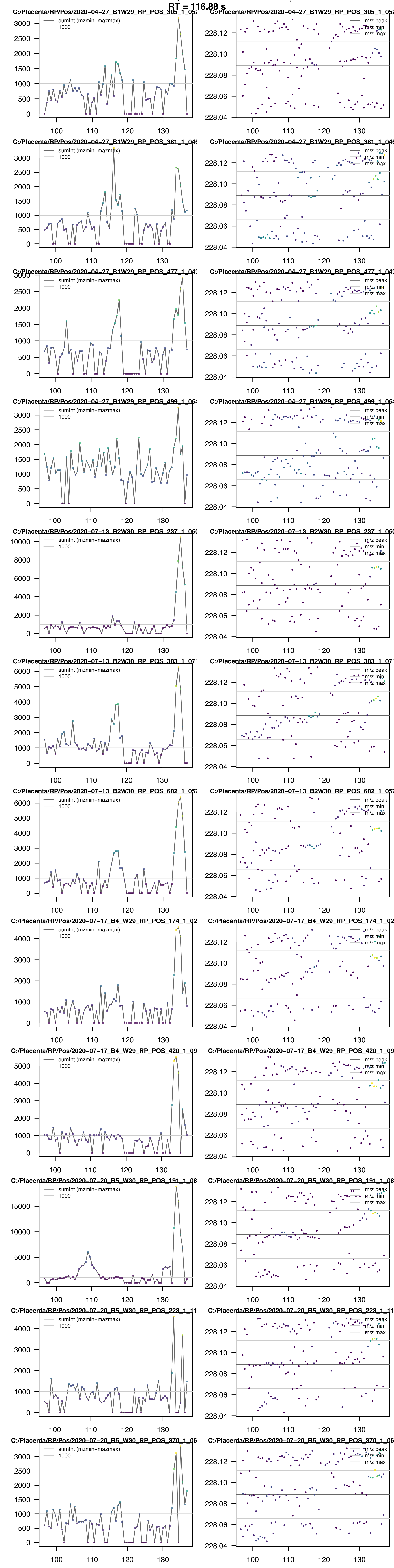

m/z 442.37125 (442.32701–442.41549)

RT = 366.12 s

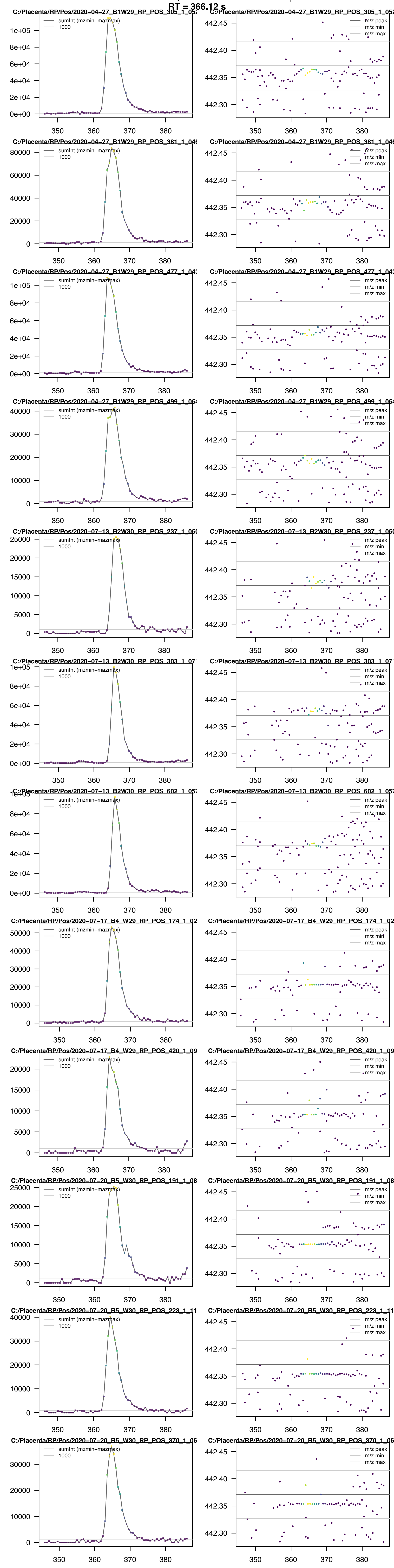

m/z 360.28867 (360.25264−360.3247)
RT = 312 s

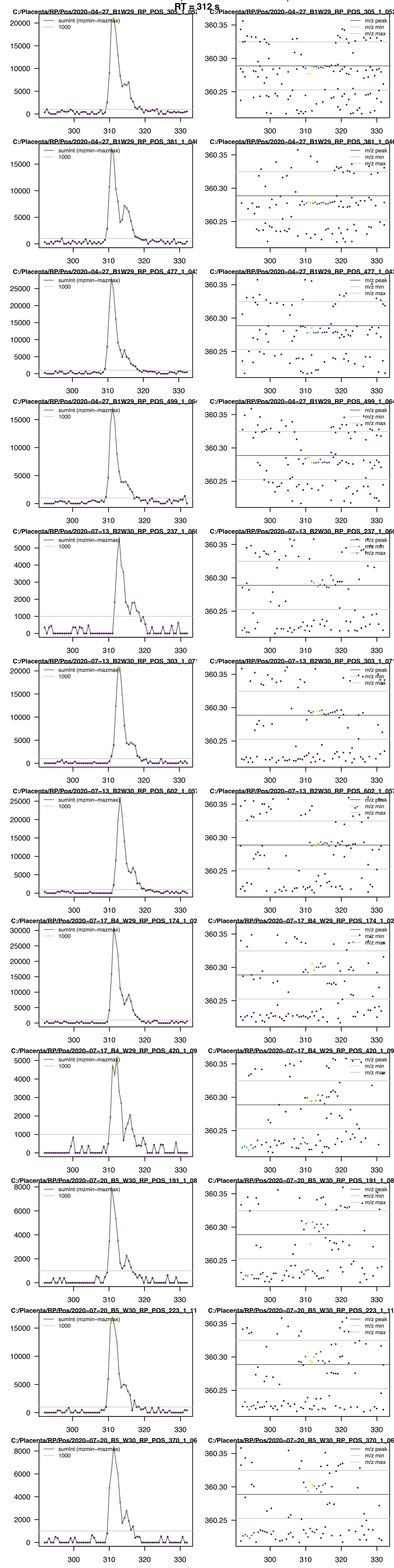

m/z 273.10336 (273.07605–273.13067)
RT = 39.84 s

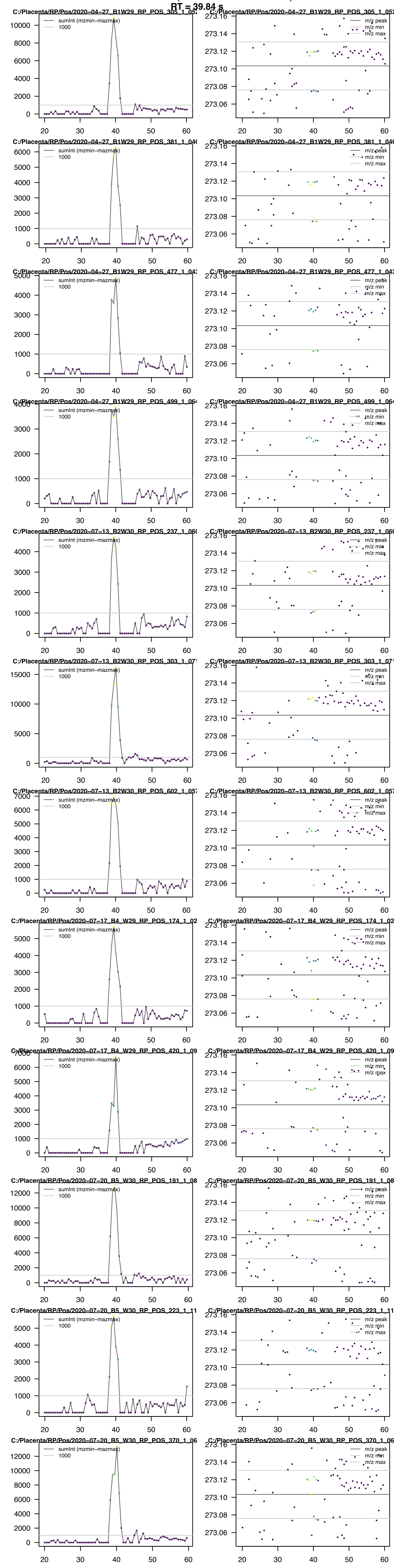

Supplement: Supplementary file 1 — Supplementary Material 1 [file 11306_2024_2092_MOESM1_ESM.pdf]
